# Evolution of Rosaceae Fruit Types Based on Nuclear Phylogeny in the Context of Geological Times and Genome Duplication

**DOI:** 10.1093/molbev/msw242

**Published:** 2016-11-17

**Authors:** Yezi Xiang, Chien-Hsun Huang, Yi Hu, Jun Wen, Shisheng Li, Tingshuang Yi, Hongyi Chen, Jun Xiang, Hong Ma

**Affiliations:** 1 State Key Laboratory of Genetic Engineering and Collaborative Innovation Center of Genetics and Development, Ministry of Education Key Laboratory of Biodiversity and Ecological Engineering, Institute of Plant Biology, Center of Evolutionary Biology, School of Life Sciences, Fudan University, Shanghai, China; 2 Department of Biology, the Huck Institutes of Life Sciences, the Pennsylvania State University, University Park, PA; 3 The Smithsonian Institution, Washington, DC; 4 Hubei Collaborative Innovation Center for the Characteristic Resources Exploitation of Dabie Mountains, Hubei Key Laboratory of Economic Forest Germplasm Improvement and Resources Comprehensive Utilization, School of Life Sciences, Huanggang Normal College, Huanggang, Hubei, China; 5 Plant Germplasm and Genomics Center, Germplasm Bank of Wild Species, Kunming Institute of Botany, Chinese Academy of Sciences, Kunming, China

**Keywords:** coalescence, fruit evolution, molecular clock, nuclear phylogeny, Rosaceae, genome duplication

## Abstract

Fruits are the defining feature of angiosperms, likely have contributed to angiosperm successes by protecting and dispersing seeds, and provide foods to humans and other animals, with many morphological types and important ecological and agricultural implications. Rosaceae is a family with ∼3000 species and an extraordinary spectrum of distinct fruits, including fleshy peach, apple, and strawberry prized by their consumers, as well as dry achenetum and follicetum with features facilitating seed dispersal, excellent for studying fruit evolution. To address Rosaceae fruit evolution and other questions, we generated 125 new transcriptomic and genomic datasets and identified hundreds of nuclear genes to reconstruct a well-resolved Rosaceae phylogeny with highly supported monophyly of all subfamilies and tribes. Molecular clock analysis revealed an estimated age of ∼101.6 Ma for crown Rosaceae and divergence times of tribes and genera, providing a geological and climate context for fruit evolution. Phylogenomic analysis yielded strong evidence for numerous whole genome duplications (WGDs), supporting the hypothesis that the apple tribe had a WGD and revealing another one shared by fleshy fruit-bearing members of this tribe, with moderate support for WGDs in the peach tribe and other groups. Ancestral character reconstruction for fruit types supports independent origins of fleshy fruits from dry-fruit ancestors, including the evolution of drupes (e.g., peach) and pomes (e.g., apple) from follicetum, and drupetum (raspberry and blackberry) from achenetum. We propose that WGDs and environmental factors, including animals, contributed to the evolution of the many fruits in Rosaceae, which provide a foundation for understanding fruit evolution.

## Introduction

Angiosperms (flowering plants) differ from all other plants by producing seeds within the fruits, which serve to protect developing seeds and also facilitate seed dispersal, by animals, wind, or water (Ridley 1930; Seymour et al. 2013). The fruit wall insulates the tender young seeds from harsh environments, protecting the seeds against damage from pathogens, water loss, and other stresses. Fruits of many plants are highly nutritious and are consumed by animals, long before there were humans; the fruits collected or incompletely eaten by animals are often transported away from the plants that produce them (Fleming and Kress 2011). Sometimes, undigested seeds are excreted by the animals and allowed to germinate subsequently (Ridley 1930). Some fruits develop appendages that attach to passing animals, and are carried elsewhere. Many plants produce fruits with extensions such as wings (as in maple) or pappus (as in Asteraceae members) that allow easy dispersal by wind. Plants like coconut produce fruits that can travel along currents to distant coasts. The protection provided by the fruits and the increased seed dispersal due to fruit characteristics have likely contributed to the unparalleled successes of angiosperms, totaling over 300,000 extant species (Fuentes and Vivian-Smith 2009; Judd et al. 2015; Spooner 2016). In addition, humans have benefited greatly from fruits of both wild and cultivated species, including many fleshy fruits, such as apple, peach, orange, melon, grapes, and date palm, but also dry fruits such as grains, beans, and chestnuts. Therefore, investigating fruit evolution is important for the understanding of the evolution of angiosperms, and can impact other fields such as ecology and agriculture.

**Fig. 1:**
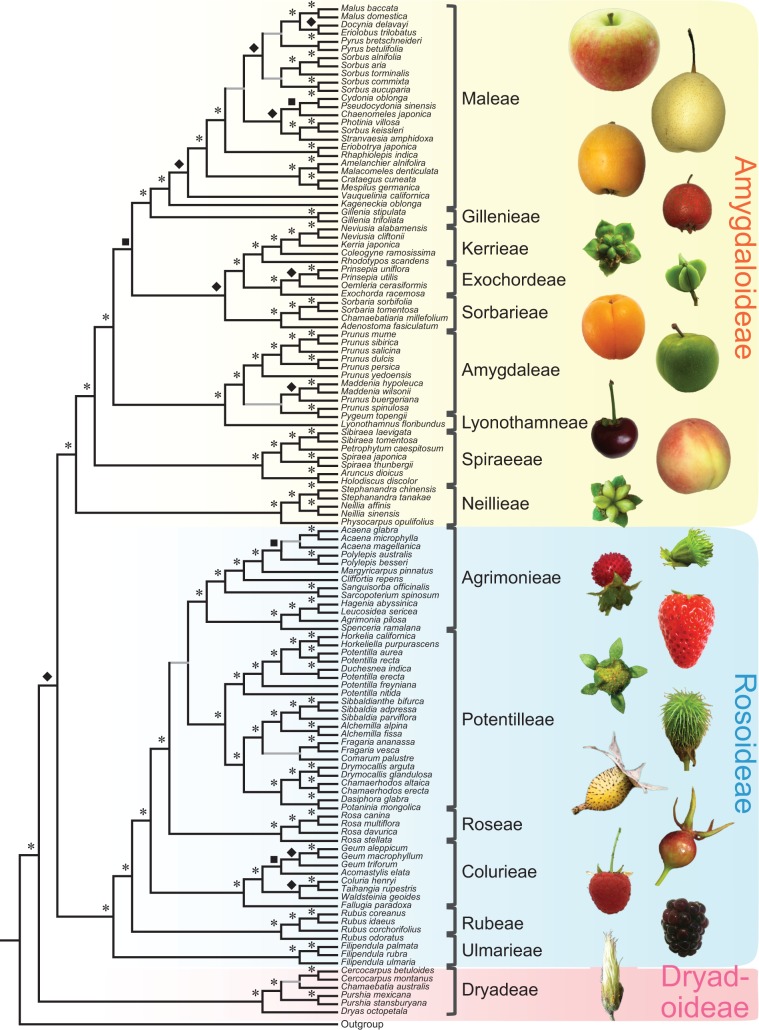
A summary of Rosaceae phylogeny and Rosaceae fruit morphologies. On the left is a summary tree with results from five coalescence analyses of 882, 571, 444, 256, and 113 gene sets, respectively, and a concatenation analysis using the 113-gene supermatrix. Topologies consistent in all six trees are drawn in black lines. Grey lines show uncertain relationships, with some trees support the topology. For further information see figure 3 and supplementary figure S14, Supplementary Material online. Asterisks (*) indicate 100% supports in all six trees. Diamonds indicate more than 90% supports in at least five trees and more than 85% supports in all six trees. Squares indicate more than 80% supports in at least three trees and more than 40% supports in all six trees. Plant photographs on the right show the diversity of Rosaceae fruits. The left row (from the top) includes *Malus pumila* (apple), *Eriobotrya japonica* (loquat), *Kerria japonica*, *Prunus armeniaca* (almond), *Prunus* sp. (cherry), *Spiraea thunbergii*, *Duchesnea indica*, *Potentilla supina*, *Rosa laevigata*, *Rubus* sp. (raspberry), and *Dryas octopetala*. The right row (from the top) includes *Pyrus bretschneideri* (pear), *Crataegus pinnatifida*, *Exochorda racemosa*, *Prunus salicina* (plum), *Prunus persica* (peach), *Agrimonia pilosa*, *Fragaria* × *ananassa* (strawberry), *Geum aleppicum*, *Rosa* sp., and *Rubus fruticosus* (blackberry).

In some angiosperm families, such as the Brassicaceae (cabbage and relatives), Fabaceae (legumes), Poaceae (grasses), and Vitaceae (grape), different species of the same family produce relatively similar fruits belonging to the same types with variation in size and shape, whereas other families contain species producing a few fruit types. In contrast, Rosaceae species produce several highly distinctive types of fruits (Potter et al. 2007a; Phipps 2014) (fig. 1), including fleshy pomes (with a relatively soft core and multiple seeds: apple, pear), drupes (with a hard central shell and a single seed: peach, plum, cherry), and dry achene (with a thin wall and a single seed). Furthermore, some species produce aggregate fruits such as drupetum (a group of tiny drupelets loosely attached to a central structure, as in raspberry), achenetum (multiple achenes from a single flower), sometimes with a fleshy enlarged receptacle (strawberry) or an enveloping hypanthium (fused lower portions of the sepals, petals and stamens, as in rose), and follicetum (several pod-like structures each with one or more seeds, from a single flower). In particular, Rosaceae members produce many economically important fruits (apple, pear, peach, plum, cherry, strawberry, and raspberry), which have increased in size and sweetness due to human domestication efforts. Therefore, Rosaceae provide an excellent system to conduct comparative and evolutionary studies of fruits.

Rosaceae is a moderately large angiosperm family in the order Rosales, with about 3000 species, 3 subfamilies, 16 tribes, and 88–100 genera (Hummer and Janick 2009; Phipps 2014). The family has a worldwide distribution, with particular diverse presence in Northern Hemisphere temperate forests, in which woody Rosaceae members are important forest trees, providing habitats and foods for birds, mammals, and other animals (Hummer and Janick 2009). In addition to important commercial fruit species, Rosaceae also include many ornamental flowers (roses, meadowsweets, hawthorns, crabapples, and rowans). Furthermore, five Rosaceae genomes have been published, including woodland strawberry (*Fragaria vesca*), domesticated apple (*Malus × domestica*), pear (*Pyrus bretschneideri*), peach (*Prunus persica*), and Mei (*Prunus mume*, related to apricot) (Velasco et al. 2010; Shulaev et al. 2011; Zhang et al. 2012b; Verde et al. 2013; Wu et al. 2013), providing important resources for comparative analyses.

Rosaceae species are classified into three subfamilies (Potter et al. 2007a), with two large ones, Rosoideae and Amygdaloideae, having ∼2000 and ∼1000 species, respectively, and a small one, Dryadoideae, with fewer than 30 species. Previously, largely according to fruit and other morphological characteristics, Rosaceae were divided into four subfamilies, Rosoideae (*s. l*., with aggregate fruits), Maloideae (= tribe Maleae/Pyreae with over 30 genera and at least 500 species including apple/pear and relatives with pomes; Maleae hereafter for simplicity), Amygdaloideae (*s. s.* = tribe Amygdaleae, with drupes), and Spiraeaoideae (Schulze-Menz 1964). Recent molecular analyses support the separation of the former Rosoideae (*s. l.*) into Rosoideae (*s. s*.) and Dryadoideae, and the combination of the previous Maloideae, Amygdaloideae (*s. s*.), and Spiraeaoideae into the current Amygdaloideae (*s. l.*) (Morgan et al. 1994; Potter et al. 2007a; Chin et al. 2014; Phipps 2014). To avoid confusion, we will use Rosoideae, Amygdaloideae and Dryadoideae in the current sense, and use the tribal names Maleae and Amygdaleae to refer to the respective groups, rather than using the older subfamily names. This chloroplast-gene based system of three subfamilies supports complex evolution of Rosaceae fruits, with multiple fruit types in each of several clades (Potter et al. 2007a); however, the unresolved relationships among tribes and genera have hindered the understanding of evolutionary history of fruit types in Rosaceae.

Importantly, the species richness of Rosaceae could be partly related to polyploidization and species radiation in the family history, with evidence for polyploidy events in the two larger subfamilies (Talent and Dickinson 2005; Vamosi and Dickinson 2006; Dickinson et al. 2007; Rousseau-Gueutin et al. 2009; Lo et al. 2010; Schmidt-Lebuhn et al. 2010; Considine et al. 2012; Burgess et al. 2014; Fougere-Danezan et al. 2015). In particular, the ancestor of Maleae was proposed to be a hybrid of the ancestors of the Spiraeoideae and the Amygdaleae, in part because all Maleae members have a base chromosome number of 17, with an exception of an early branching genus *Vauquelinia* having a base chromosome number of 15, whereas the putative parents have chromosome number of 9 and 8 for Spiraeoideae and Amygdaleae, respectively (Robertson et al. 1991). More recently, an alternative hypothesis was proposed that Maleae originated with a whole-genome duplication (WGD) event in a relative of the ancestor of *Gillenia* (*x* = 9) (Evans et al. 2000; Evans and Campbell 2002; Velasco et al. 2010; Verde et al. 2013). However, the possible occurrence and timing of other WGDs are unclear. Therefore, investigation of the polyploidy/WGD events will contribute to the understanding of Rosaceae evolution.

Both the study of fruit evolution and analysis of WGDs depend on a well-resolved phylogeny of Rosaceae. Specifically, the subfamily Amygdaloideae alone has multiple tribes, with different fruit types, such as pome, drupe, and follicetum; however, the phylogenetic relationships among the tribes are unclear (Potter et al. 2007a). In the first molecular phylogenetic study of Rosaceae based on *rbcL* sequences, Maleae (e.g., apples and pears), Amygdaleae (e.g., peaches and plums), and Rosoideae (e.g., roses, strawberries, raspberries, and others) are monophyletic clades but the previous Spiraeoideae were not monophyletic, with several distinct clades (Morgan et al. 1994). Additional phylogenetic studies have provided valuable information for subgroups in Rosaceae, including the tribes Maleae, Amygdaleae, Spiraeeae, and Potentilleae, and the genera *Geum*, *Potentilla, Rosa*, *Rubus, Neillia*, and *Prunus* (Alice and Campbell 1999; Lee and Wen 2001; Smedmark and Eriksson 2002; Aldasoro et al. 2005; Campbell et al. 2007; Potter et al. 2007b; Gehrke et al. 2008; Lundberg et al. 2009; Dobes and Paule 2010; Lo and Donoghue 2012; Oh 2013; Chin et al. 2014; Zhu et al. 2015). However, relationships among Rosaceae tribes and genera remain unclear, in part because of the polyploidy events and rapid separation/diversification among some clades.

In recent years, phylogenetics using dozens or more nuclear genes have been successful for reconstructing phylogeny of difficult angiosperm groups with rapid radiations, such as those of five major angiosperm lineages, of Brassicaceae, and of Caryophyllales (Zhang et al. 2012a; Zimmer and Wen 2013; Zeng et al. 2014; Yang et al. 2015; Huang et al. 2016). The rapid advances of high-throughput sequencing technologies have greatly facilitated transcriptome sequencing, allowing numerous phylogenetic markers to be identified (Wen et al. 2015; Zimmer and Wen 2015). Furthermore, nuclear genes provide information from bi-parental inherence and allow the detection of WGDs and other events. To reconstruct the Rosaceae phylogeny, we sequenced 123 transcriptomes (115 Rosaceae species and 8 other Rosales species), representing all Rosaceae tribes and nearly all multi-species genera. Multiple datasets of low-copy putative orthologous genes were identified and then used for phylogenetic reconstruction, resulting in a highly supported Rosaceae phylogeny, which is consistent with well resolved relationships obtained previously and presents newly resolved relationships. The nuclear gene datasets were also used for molecular clock estimates of the origin of the crown Rosaceae at the boundary of Early and Late Cretaceous (∼100 Ma), and those of the subfamilies and major tribes (between Late Cretaceous to Eiocene), providing possible geological timeframe and climate conditions for the diversification of tribes and genera. In addition, ancestral character reconstructions of fruit types and other morphologies were performed, allowing the proposal of the origins and histories of multiple fleshy fruit types from dry fruit types. Specifically, drupes evolved from follicetum at least twice, once for *Prunus* (peach, plum and their relatives) and the other for *Prinsepia*; pomes (apple, pear, and other fleshy-fruited members of Maleae) evolved from coccetum, which was in turn derived from follicetum; multiple drupelets from the same flower also evolved at least twice, from follicetum (nuculanium, for *Rhodotypos*, with a few drupelets) and from achenetum (drupetum, for *Rubus*, with many drupelets). The newly resolved Rosaceae phylogeny also allows the placement of WGD events, including two near the origin of Maleae, presenting a comprehensive understanding of Rosaceae fruit evolution in the context of dramatic genome changes and geological times with changing environments.

## Results and Discussion

### Taxon Sampling and Transcriptomes for Gene Marker Identification

In this study, a total of 124 Rosaceae species were included (supplementary table S1, Supplementary Material online), covering all three subfamilies (6 Dryadoideae, 54 Rosoideae, 64 Amygdaloideae) and 16 tribes in Rosaceae, as well as nearly all multi-species genera, which represented more than 98% of Rosaceae species. For genera that were reported to be nonmonophyletic (*Potentilla* and *Sorbus*), species representing more than one clade were sampled. For tribes (Roseae, Rubeae, and Ulmarieae) with only one genus and uncertain relationships with other tribes, more than one species were sampled. In addition, 24 other angiosperm species were used as outgroups (supplementary table S1, Supplementary Material online), including 8 for other families in Rosales and 7 in Fagales, an order relatively close to Rosales within Rosids, as well as others from four orders (Saxifragales, Caryophyllales, Ranunculales, and Laurales) with varying evolutionary distances from Rosaceae (The Angiosperm Phylogeny Group 2016).

Among the 124 Rosaceae datasets, 115 were new transcriptome datasets generated for this study (6 Dryadoideae, 50 Rosoideae, and 59 Amygdaloideae) (supplementary table S2, Supplementary Material online); for outgroups, transcriptomes for 8 species in other Rosales families were also generated (supplementary table S2, Supplementary Material online). For these transcriptomes, young leaves and/or floral buds and/or fruits were sampled and used for total RNA isolation. Transcriptomes with 11,057,450–102,770,184 reads were obtained using the Illumina technologies and the reads for each species were assembled into 20,532–67,177 contigs of cDNA sequences (supplementary table S2, Supplementary Material online). The contigs have median lengths of 726–1632 nucleotides, with N50 values ranging from 510 to 1095 nucleotides. In addition to the new transcriptomes, five genome sequence datasets and two Sequence Read Archive transcriptome datasets were retrieved for Rosaceae species from public databases. Moreover, newly generated shotgun genomic sequences for two Rosoideae species (*Cliffortia repens* and *Hagenia abyssinica*) were also included.

### Selection of Low-Copy Candidate Orthologous Nuclear Genes

Low-copy candidate orthologous genes were identified for use as phylogenetic markers (for details see “Materials and Methods” section). To avoid possible biases of specific gene sets, candidate marker genes were identified using two approaches. The first approach began with a set of 931 candidate orthologous genes previously identified for a study of an angiosperm deep phylogeny (Zeng et al. 2014) (see “Materials and Methods” section), with low copy nuclear genes shared by nine angiosperm species (*Arabidopsis thaliana, Populus trichocarpa, Glycine max, Medicago truncatula, Vitis vinifera, Solanum lycopersicum, Oryza sativa, Sorghum bicolor*, and *Zea mays*), among which *Arabidopsis thaliana*, *Populus trichocarpa, Glycine max*, and *Medicago truncatula* represent taxa in Rosids. These 931 genes were then filtered using (1) a minimal length of 1000 base pairs (bps) in four sequenced genomes in Rosaceae and an outgroup species and (2) the presence in six or more of 10 selected taxa (eight Rosaceae members plus two outgroup species), yielding 546 orthologous groups (OGs) (see “Materials and Methods” section).

It was reported that Rosaceae members had undergone polyploidization events (Vamosi and Dickinson 2006; Zhao et al. 2016), resulting in many duplicate genes. If the duplicate genes subsequently returned to single-copy status due to independent losses of distinct paralogues in different lineages, the remaining genes are referred to as hidden paralogues. To avoid such hidden paralogues, we constructed gene phylogenies of the above-mentioned 546 OGs using sequences from eight representative Rosaceae species with well-supported relationships and two outgroup species (see “Materials and Methods” section). The OGs with single gene trees whose well-supported topologies were not contradictory to the known species relationships were retained, yielding 407 candidate orthologous genes.

Our second approach started from a set of 3863 single copy genes shared by three Rosaceae species with sequenced genomes (*Fragaria vesca, Prunus persica*, and *Prunus mume*) and *Cucumis sativus* in Cucurbitaceae, a family closely related to Rosaceae. To avoid selecting the genes from the first approach again, those in the previous set were not considered further in this approach. After filtering with minimal length of 1000 bps in genes from *Cucumis sativus*, 2124 genes were retained. Considering that Maleae has undergone at least one recent lineage-specific WGD, marker genes with only one or two copies in *Malus × domestica* and *Pyrus bretschneideri* were retained from among the 2124 genes, ultimately yielding 475 genes. The two approaches yielded a total of 882 genes (supplementary table S3, Supplementary Material online); when these genes were used to reconstruct the Rosaceae phylogeny using the coalescence method (see below), all subfamilies and tribes were monophyletic.

Because much of the molecular phylogenetic analysis has been performed using concatenation of multiple genes (supermatrices) and such datasets are needed for molecular clock estimates, we also wanted to use this approach. It has been argued that the use of supermatrices of hundreds of genes with methods such as maximum likelihood (ML) could produce highly supported but wrong topologies (Seo 2008). Furthermore, the use of relatively small numbers of genes can save computational time and facilitate the inclusion of additional taxa. Thus, selection of subsets of genes were made by examining gene tree topologies for hidden paralogues using memberships in the same order (including outgroups), Rosaceae subfamily, and tribe, successively trimming to subsets of 571, 444 and 256 genes, respectively (see “Materials and Methods” section). For even a smaller subset, further selections were made using topological information of four groups (see “Materials and Methods” section) within Maleae to avoid possible hidden paralogues, resulting in 113 genes, which are less prone to systematic errors when using maximum likelihood analyses with supermatrices. In addition, among the 475 genes from the second approach, 163 genes were selected after eliminating hidden paralogues using gene trees from 31 species, including 25 in Maleae and 6 other Rosaceae members, for relationships within Maleae (see “Materials and Methods” section).

### A Well Resolved Rosaceae Phylogeny Supported by Multiple Analyses

**Fig. 2:**
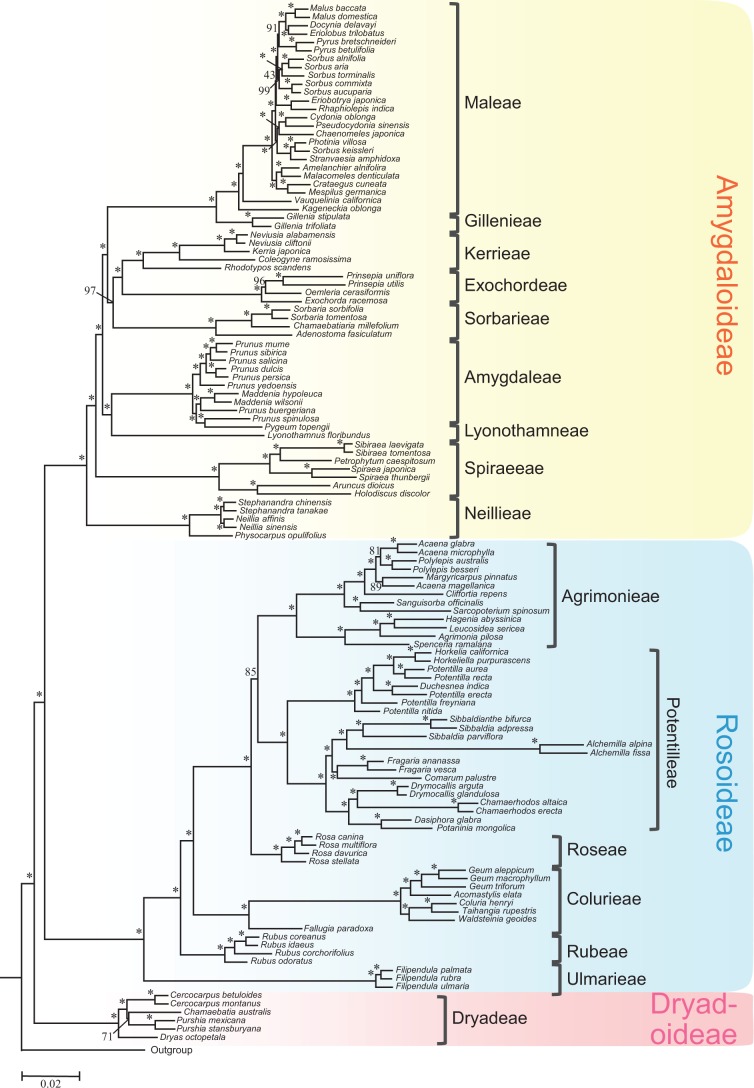
A phylogeny from ML analysis using a dataset of concatenated 113 gene sequences. Numbers associated with nodes indicate bootstrap values obtained by Maximum Likelihood (ML) analyses. Asterisks (*) indicate 100% support.

To reconstruct the Rosaceae phylogeny, we used multiple datasets to minimize possible biases of specific genes. First, we used the combined 882-gene set with the coalescence method implemented in ASTRAL v4.4.4 (Mirarab et al. 2014), yielding a tree with strong support for most nodes (fig. 1; supplementary fig. S1, Supplementary Material online). Largely consistent results were also obtained using the coalescence method with the 571, 444, 256, and 113 gene sets (fig. 1; supplementary figs. S2–S5, Supplementary Material online). A maximum likelihood (ML) analysis was also performed with the 113-gene supermatrix (fig. 2), because the concatenation method with relatively small number of genes has less risk of systemic errors than those using much larger numbers of genes, such as several hundreds. The relationships for the vast majority of taxa in the trees from the six analyses are consistent, as summarized in figure 1, although there are some differences between the trees for a few taxa (see below for more details).

**Fig. 3:**
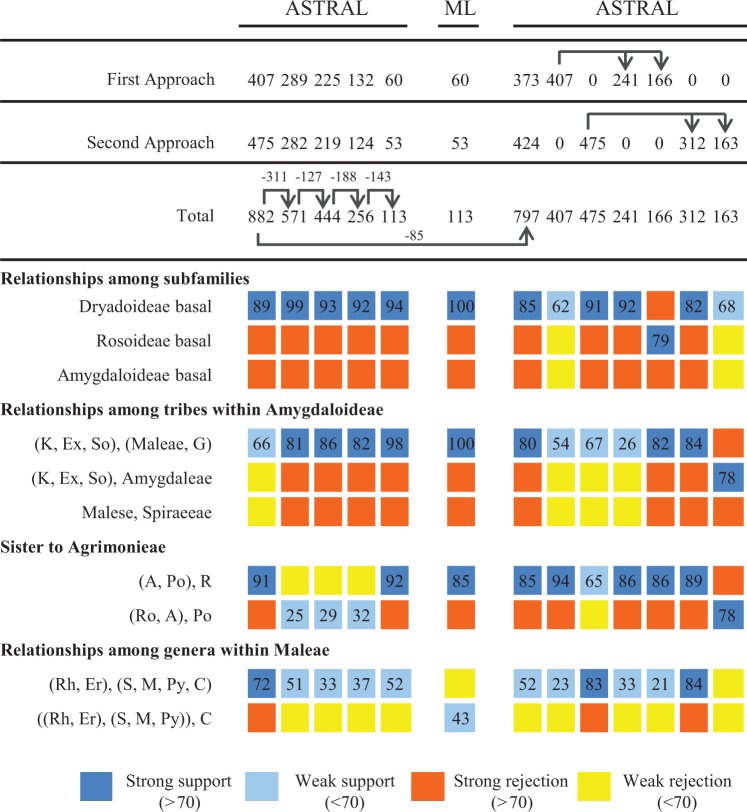
A summary of alternative topologies in results from 12 coalescence analyses and one concatenation analysis. At the top are phylogenetic methods and numbers of genes in various sets and relationships between gene sets (see “Results” and “Materials and Methods” sections, and supplementary fig. S24, Supplementary Material online for additional information). The column on the left indicates possible topologies. Designations: Dryadoideae basal: Dryadoideae at the basal position, as sister to the combined clade of Amygdaloideae and Rosoideae, others similarly. K: Kerrieae. Ex: Exochordeae. So: Sorbarieae. G: Gillenieae. A: Agrimonieae. Po: Potentilleae. Ro: Roseae. Rh: *Rhaphiolepis*. Er: *Eriobotrya*. S: a combined clade of *Sorbus alnifolia*, *Sorbus aria*, *Sorbus torminalis*, *Sorbus commixta*, and *Sorbus aucuparia*. M: a clade including *Malus baccata* and its three nearest relatives (as shown in fig. 1). Py: *Pyrus*. C: *Cydonia* and its five nearest relatives (as in fig. 1). Number in each square refers to values in support of a particular topology indicated in the left column. Strong support refers to support values of at least 70%. Weak support refers to values less than 70%. If there is a strong support for a topology in a particular node, other topologies at this node are strongly rejected. If there is a weak support for a topology in a particular node, other topologies at this node are weakly rejected.

We also obtained a 797-gene dataset after removing some genes that placed outgroup species within a clade of Rosaceae species and used these genes to produce a phylogeny with the coalescence method (supplementary fig. S6, Supplementary Material online). In addition, we reconstructed phylogenies using the 407 (supplementary fig. S7, Supplementary Material online) and 475 (supplementary fig. S8, Supplementary Material online) genes obtained from the first and the second approaches, respectively. To test the effectiveness of smaller gene sets, we have divided the 407 (into 241 and 166 genes) and 475 (into 312 and 163 genes) genes into smaller sets and used them for additional phylogenies (supplementary figs. S9–S12, Supplementary Material online). These phylogenies are largely consistent with that shown in figure 1, but do not always support those relationships that are still somewhat uncertain (fig. 3; see below for more details and discussion).

In agreement with the previous molecular phylogenetic studies, all three subfamilies and 16 tribes form monophyletic clades with 100% support in each of the thirteen topologies (fig. 2; supplementary figs. S1–S12, Supplementary Material online). Importantly, our results strongly support the hypothesis that Dryadoideae is the basal clade of Rosaceae with more than 90% support in five topologies, more than 85% support in all six topologies for figure 1; however, Rosoideae was inferred in a previous study to be the sister lineage to the combined clade of the other two subfamilies (Potter et al. 2007a). To test whether the hypothesis of Dryadoideae being basal is supported by various subsets of marker genes, we examined all 13 topologies (fig. 2, supplementary figs. S1–S12, Supplementary Material online) and summarize the results in figure 3. Among the 13 trees, 10 strongly supported and two weakly supported Dryadoideae as the sister to the remainder of the family, only one, with 166 genes, supports Rosoideae being basal (fig. 3). One possibility was that different gene sets contained genes with different topologies. Detailed examination of single-gene trees revealed that, in the 241, 312, and 163 gene sets, the numbers of genes supporting (with more than 50 bootstrap value) Dryadoideae as sister of the combined clade of Rosoideae and Amygdaloideae are 74, 91, and 47, respectively; these numbers are larger than that of genes supporting Rosoideae (55, 85, and 38) or Amygdaloideae (48, 66, and 38) as the basal clade (supplementary fig. S13, Supplementary Material online). On the other hand, in the 166-gene set, 46 genes support Rosoideae as the basal clade, only slightly more than 39 and 42 genes, respectively, for Dryadoideae or Amygdaloideae being basal. Therefore, there were greater numbers of single-gene trees supporting Dryadoideae being first lineage to separate, and variation in the number of genes with specific topologies could at least provide partially explanation for the difference among the results from different gene sets.

Within Dryadoideae our sampling included four genera, with strong support for *Dryas* being the basal genus, in agreement with previous results (Potter et al. 2007a). Also, *Purshia* is sister to the combined clade of *Cercocarpus* and *Chamaebatia*, with strong supports from most of the analyses (supplementary fig. S14, Supplementary Material online). Within Rosoideae, the tribes Ulmarieae, Rubeae, and Colurieae occupy the basalmost and the next two basal positions, respectively; these relationships are largely consistent with previous results that they are the three successive basalmost lineages, although the relatively positions of Rubeae and Colurieae were uncertain previously (Potter et al. 2007a). The remaining three tribes Roseae, Potentilleae, and Agrimonieae form a strongly supported clade (fig. 1), and support values for Roseae being the basal lineage in this clade were strong in eight of the 13 analyses (fig. 3). The relationships between genera in Rosoideae were also very well resolved (fig. 1). To test whether the process of filtering orthologous genes from 882 to 113 has removed paralogous genes supporting a particular topology, we have examined the position of Roseae, Potentilleae, and Agrimonieae in each single gene tree with more than 70% bootstrap value in each gene set (supplementary fig. S13, Supplementary Material online). In the 882-gene coalescence tree (fig. 3, supplementary fig. S1, Supplementary Material online), Roseae is highly supported as the basal clade; this is consistent with the number of single gene trees supporting Roseae being basal (RB) vs. Potentilleae being basal (PB) (183 vs. 173). After filtering from 882 to 571, the numbers of single-gene trees supporting RB was reduced by 63, greater than 55, the number reduced for PB. Among the remaining 571 genes, the numbers of gene trees supported RB (120) and PB (118) were nearly identical, in agreement with the weak support for PB in the 571 coalescence tree (fig. 3, supplementary fig. S2, Supplementary Material online). After other filtering processes, in the 113 gene set, the single-gene trees supporting RB and PB were 36 and 24, respectively, with a ratio of 3 to 2 (supplementary fig. S13, Supplementary Material online), consistent with the 113-gene coalescence tree strongly supporting RB (fig. 3, supplementary fig. S5, Supplementary Material online). In short, the changes in number of genes supporting specific topologies might be one reason for the differences between the coalescence trees generated using different gene sets.

In the subfamily Amygdaloideae, there are nine tribes and the relationships for several of them were uncertain previously (Potter et al. 2007a). From our results, the tribe Neillieae with follicetum fruit type is maximally supported in all 13 topologies as the basalmost lineage of Amygdaloideae, followed by the tribe Spiraeeae with follicle fruit (100 support value in 12 trees, and 97 in one) (fig. 2, supplementary figs. S1–S12, Supplementary Material online). Lyonothamneae (with a single species), which was previously placed as the basal lineage of the subfamily, is now strongly supported (100 support value in 11 trees, with 99 and 94, respectively, in the other two) as sister to Amygdaleae (peach, plum, and cherry); they together occupy the third basal position in the subfamily. Next along the backbone is a clade with three tribes, with Sorbarieae being sister to the combined clade of Exochordeae and Kerrieae. The close relationship of Exochordeae and Kerrieae was also supported previously (Potter et al. 2007a). Finally, the fleshy-fruited clade Maleae and dry-fruited Gillenieae are sisters with 100% support in all 13 topologies for figure 2 and other phylogenies (supplementary figs. S1–S12, Supplementary Material online). In addition, the sister relationship of the [Sorbarieae, (Exochordeae, Kerrieae)] and (Maleae, Gillenieae) clades is strongly supported in eight of the thirteen topologies, and weakly supported in four others (fig. 3). Within Maleae, *Kageneckia* and *Vauquelinia* occupy the successive basal positions, with a highly supported clade of four genera (*Amelanchier*, *Malacomeles*, *Crataegus*, and *Mespilus*) being the next basal group (fig. 1). These four genera were also found in a well-supported clade previously, but the relationships of this clade with other genera of Maleae were not clear (Potter et al. 2007a; Lo and Donoghue 2012). The remaining 12 genera sampled here for Maleae form a large and very well supported clade, including several smaller clades that are each well-supported (fig. 1), with support for the clade of *Eriobotrya* and *Rhaphiolepis* being sister to the clade with the remaining 10 genera (fig. 3). As presented below, near the origin of Maleae there were two successive events of whole genome duplication, making the complex history of this tribe difficult to resolve, with some relationships requiring further analyses (supplementary fig. S14, Supplementary Material online).

Because several relationships are supported by some, but not all, of the analyses here, we then performed approximately unbiased (AU) test to examine the robustness of the relevant results, in two phases (Shimodaira and Hasegawa 2001). The first was to verify the relationships among three subfamilies and the tribes in Amygdaloideae (supplementary table S4, Supplementary Material online), with the topology using the 113-gene supermatrix (supplementary fig. S6, Supplementary Material online) and 20 alternative topologies. All 20 alternative topologies were rejected (*P* ≤ 0.05), and only the topology of 113-gene supermatrix analysis was not rejected, with a notably high *P* value of 0.998, supporting Dryadoideae as the basal clade of Rosaceae and the sister relationship of the Kerrieae–Exochordeae–Sorbarieae clade and the Maleae–Gillenieae clade. In contrast, the topologies with Rosoideae as the basal lineage had *P* values of 0.001 or less. The alternative topology of (Kerrieae–Exochordeae–Sorbarieae, Amygdaleae) and Maleae–Gillenieae being sisters had a *P* value of only 0.005. In the second phase, with the relationships among subfamilies and tribes fixed, the hypothesis from the 113-gene supermatrix (supplementary fig. S6, Supplementary Material online) and 47 alternative topologies for various relationships within individual tribes were analyzed (supplementary table S5, Supplementary Material online). Again the topology of 113-genes concatenation analysis has the highest *P* value of 0.820, and only four other topologies could not be rejected (*P* ≥ 0.05; AU test), including sister relationship of any two genera among *Cercocarpus*, *Chamaebatia*, and *Purshia* in Dryadoideae and Roseae, instead of Potentilleae, being sister to Agrimonieae. Hybridizations, incomplete lineage sorting and other possible factors might contribute to these uncertainties of current topology (Johnson and Soltis 1998; Wendel and Doyle 1998; Philippe et al. 2005; Jeffroy et al. 2006; Avise and Robinson 2008); future analysis with more taxon sampling in these groups might be needed to resolve their relationships.

### Rosaceae Likely Originated near the Boundary of Early to Late Cretaceous

To estimate ages of Rosaceae lineages, we used sequences from all 124 of the Rosaceae species in this study and 24 additional outgroups for a total of 148 species with 19 fossil calibrations, including 13 belongs to Rosaceae. We used the tree topology of figure 1 and the sequence matrix of the 113 genes for the estimation inferred by penalized likelihood method implemented using r8s (Sanderson 2003). Assignments of the fossil constraints are provided in the “Materials and Methods” section and supplementary table S6, Supplementary Material online.

**Fig. 4:**
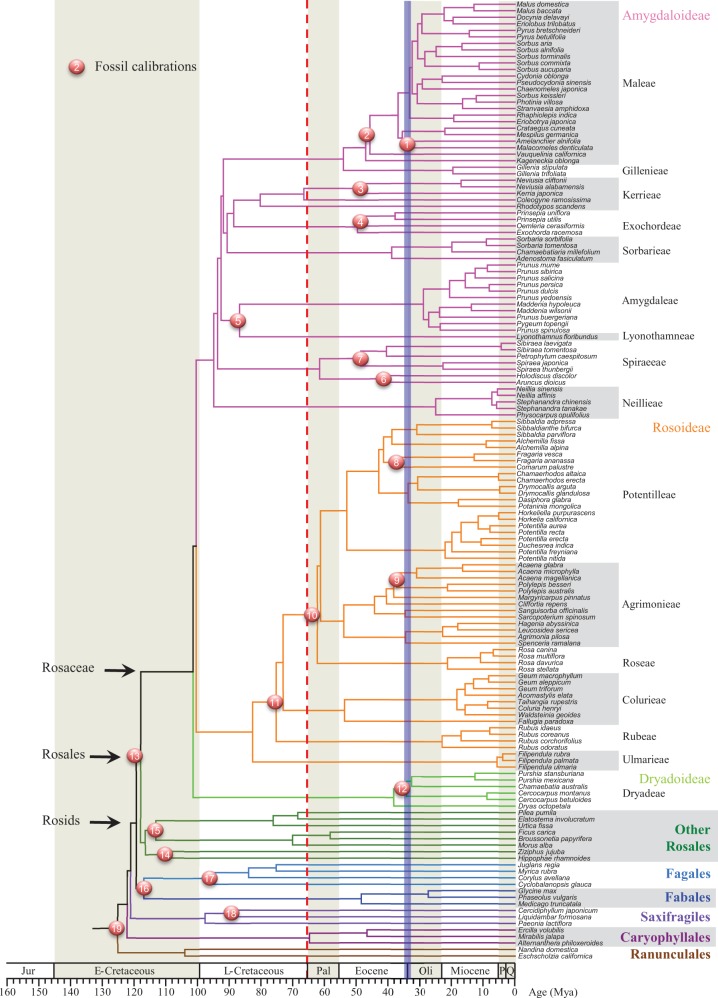
Chronogram of Rosaceae using 148 species phylogeny with 19 fossil constraints. Positions of the fossil calibrations are depicted with numbered circles. Crown nodes of Rosids, Rosales and Rosaceae are indicated with arrows. The red dashed line and blue strip highlight the Cretaceous-Paleogene and Eocene-Oligocene boundaries, respectively. Ages are presented as millions of years (Myr). There are two possible topologies within Dryadoideae (relationships among *Purshia*, *Cercocarpus* and *Chamaebatia*) having similar support from our analyses of various gene sets (see supplementary fig. S14, Supplementary Material online); here we only present the result from the analysis with the 113-gene concatenation. Jur: Jurassic. E- and L-Cretaceous mean Early and Late Cretaceous. Pal: Paleocene. Oli: Oligocene. P: Pliocene. Q: Quaternary.

The age of crown Rosaceae in our estimation was about 101.6 Ma with the separation of Dryadoideae, followed by an immediate divergence of the two largest subfamilies at 100.7 Ma (fig. 4). Töpel et al. (2012) also performed divergence time estimation using two chloroplast sequences along with eight fossil constraints and one secondary calibration. They used 10 plastid and two nuclear genes and seven fossil calibrations and set a uniform prior with a maximum age of 115 Ma for all calibration points, and then calibrated stem Rosales to 104–115 Ma according to Wang et al. (2009), resulting in an age of crown Rosaceae being slightly older than 100 Ma, very close to our result. Our age predates the result of a previous study (Chin et al. 2014), which used one secondary calibration as the fixed age of crown Rosales as well as six fossil calibrations according to a phylogeny using four chloroplast genes and the ITS sequence. They found the age of crown Rosaceae to be 90.8–85.7 Ma using the minimum (90 Ma) and maximum age (96 Ma) of crown Rosales reported by Wang et al. (2009) as two sets of fixed age constraints, which limited the age estimates. Nevertheless, all the dating results indicate that Rosaceae originated around the boundary between Early and Late Cretaceous and that the three subfamilies diverged within a short period.

After the split of the three subfamilies, there was a rapid series of nested branching of seven tribes of Amygdaloideae (∼95–90 Ma; fig. 4) during the early portion of Late-Cretaceous; on the contrary, the two most derived tribes, Maleae and Gillenieae, separated at ∼54 Ma just after the Paleocene–Eocene boundary, exhibiting a lag of ∼38 Ma from the split at ∼92 Ma from the clade of Kerrieae–Exochordeae–Sorbarieae. On the other hand, divergences of Rosoideae tribes were gradual, spanning from the middle of Late-Cretaceous (∼82 Ma) to Mid-Paleocene (∼62 Ma). In addition, there was a very long delay of ∼63 Ma before the separation of extent Dryadoideae lineages at ∼38 Ma in late Eocene, possibly due to extinction of early branches of this subfamily. Among the three largest tribes having over 500 species, Rubeae diverged earlier at 75 Ma in Late Cretaceous; the other two tribes originate under relatively stressful conditions than current environment: Potentilleae during Paleocene shortly after a global catastrophe at the boundary of Cretaceous and Paleocene (66 Ma), and Maleae during the hottest and most humid period in the Cenozoic Era.

### Evidence for Multiple Whole-Genome Duplication Events across Rosaceae with Two Close Events Shared by Members of Maleae

Genome sequences of Rosaceae members suggested that this family might have experienced one or more whole-genome duplications (Dickinson et al. 2007; Velasco et al. 2010; Wu et al. 2013; Chin et al. 2014; Zhao et al. 2016), but the timing of such event was not clear. WGD was also proposed for Maleae based on the chromosome number (Vamosi and Dickinson 2006; Dickinson et al. 2007), but there has not been support from phylogenomic analyses. To address these questions, we used a phylogenomic analysis to detect potential WGD events, as was used in several recent studies (Jiao et al. 2011, 2012; Cannon et al. 2015; Li et al. 2015; Yang et al. 2015) and recognized as an effective method (Kellogg 2016). The basic rationale is to construct gene trees on a transcriptome-wide scale and then to compare gene tree topologies with that of the organismal tree, thereby mapping duplications in each gene family tree onto the organismal tree; the detection of large numbers of gene duplications at specific nodes on the organismal tree is then seen as evidence for WGD events. We also examine the topologies of the gene tree at duplication event to evaluate the strength of support. For a node in the organismal tree with three or more species, we classified the observed topologies into three types regarding the gene retention in each of the duplicated subclades following the node of interest: type I retains both gene copies in both the large and small subclades, whereas type II and III lack duplicates for the entire small or large subclades, respectively. Among them, the type I topology provide stronger evidence than the other two types due to the presence of more genes to infer a correct phylogeny.

**Fig. 5:**
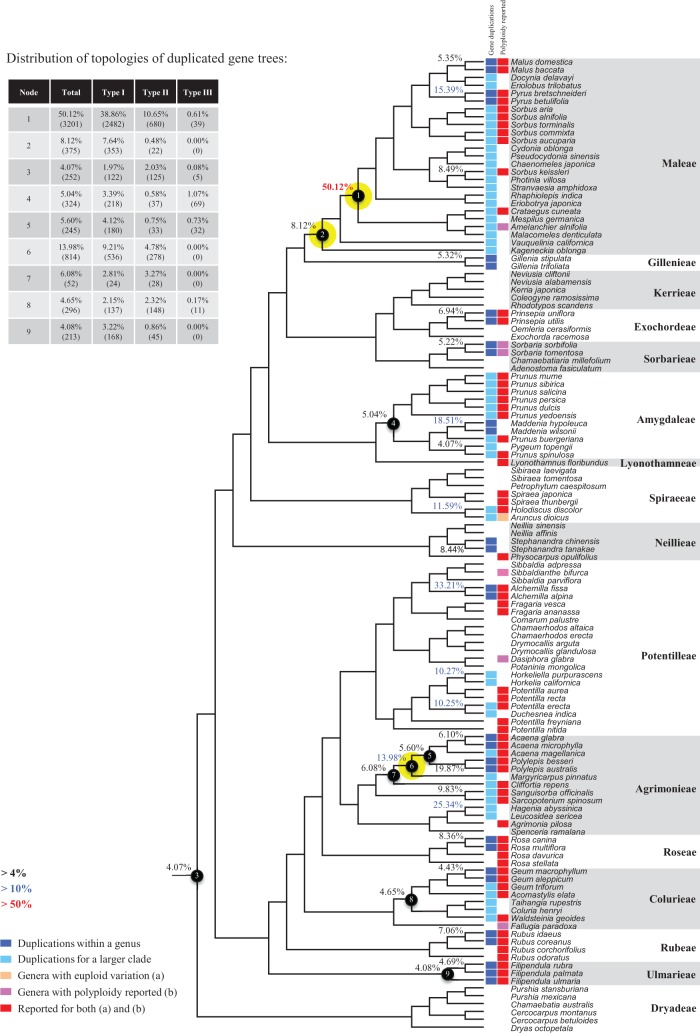
Detecting and positioning large-scale gene duplications by comparing gene family trees to the species tree. Percentage of duplicated gene families (among those containing genes from both species lineages from the node of interest) are presented adjacent to each node when there were higher than 4% gene families duplicated at the corresponding node. Numbers higher than 10% and 50% are highlighted with blue and red, respectively. Distributions of three possible topologies for each of the duplicated gene trees are shown in the upper left table, with both the percentage and the actual numbers of gene families recorded. By the diagnosis of tree topologies, nodes with strong evidence for WGD are marked with yellow. Colored boxes indicate species potentially having gene duplications within a genus (dark blue) or shared across genera (light blue); genera having euploid variations summarized by Dickinson et al. (2007) (orange) or polyploid species observed by Vamosi and Dickinson (2006) (pink) or both (red) are also highlighted.

Our results identified evidence for a duplication event (fig. 5, node 2) shared by all Maleae members with 8.12% (375 pairs) gene families showing duplications here and 7.64% (353 pairs) with strong support (type I). Much stronger signal was detected for a WGD event (node 1) shared by most of the members of Maleae except *Vauquelinia* and *Kageneckia*, supported by 50.12% (3201 pairs) gene families duplicated at this node with 38.86% having type I topology. Duplication events associated with members of Maleae have been reported previously. Based on analysis with *Malus × domestica* genome, Velasco et al. (2010) reported a WGD dated 30–45 Ma when the age for the split of grape and rosids were fixed at 115 Ma. Wu et al. (2013) reporting the genome sequence of a pear (*Pyrus bretschneideri*) also observed a WGD shared by pears and apple but not strawberry. Our findings support two possible WGD events in succession near the origin of Maleae, with the large number of resulting new gene copies as materials for functional divergences and innovations. In addition, evidence for polyploids was also found previously within Maleae for members of genera *Sorbus*, *Crataegus*, and *Amelanchier* (red boxes, fig. 5), all included in the clade derived from node 1. In addition, a recent WGD might have happened within *Pyrus*, with 15.39% (585 pairs) gene families duplicated before separation of *P. bretschneideri* and *P. betulifolia*. There was also a likely WGD event at crown node of Amygdaleae, which include members of *Maddenia* and *Pygeum*, with 5.04% (324 pairs) gene duplications and 4.12% (180 pairs) in type I gene trees. In the subfamily Rosoideae, we also detected possible WGDs within Agrimonieae, shared by *Acaena*, *Polylepis*, and *Margyricarpus* (nodes 5 and 6). Although 6.08% of gene families were duplicated at node 7, they account for only 52 paralogue pairs and do not provide strong support for WGD. Further, WGD events might have occurred for the tribes Colurieae, with support of 4.65% (296 pairs) of gene trees, and Ulmarieae with 4.08% (213 pairs) of gene trees, consistent with previous reports of polyploidy of these groups (Smedmark and Eriksson 2002; Vamosi and Dickinson 2006). In addition, 4.07% (252 pairs) of gene trees supported a possible WGD shared by all Rosaceae members, in agreement with previous proposal of paleopolyploidy of Rosaceae (Vamosi and Dickinson 2006; Dickinson et al. 2007).

Additional putative WGDs with more than 10% supporting gene trees were detected in more recent lineages of one or two genera, including the clades of *Holodiscus* and *Aruncus*, of *Horkeliella* and *Horkelia*, of *Hagenia* and *Leucosidea*, of *Potentilla erecta* and *Duchesnea indica*, and also the clades of genera *Maddenia* and *Alchemilla*. Among these potential events, *Holodiscus*, *Aruncus*, and *Alchemilla* were reported to have polyploids (Mishima et al. 2002; Gu et al. 2003; Vamosi and Dickinson 2006; Dickinson et al. 2007; Phipps 2014), supporting our hypotheses. More nodes with 10% to 4% duplicated gene families can also be observed, including those of genera *Malus*, *Gillenia*, *Prinsepia, Sorbaria, Maddenia, Stephanandra, Acaena*, *Polylepis*, *Rosa*, *Geum*, and *Rubus*. Although these percentages are not high, polyploids have been found in many of these genera, except *Gillenia* and *Stephanandra* (Gu et al. 2003; Vamosi and Dickinson 2006; Dickinson et al. 2007; Phipps 2014).

### Ancestral State Reconstruction for Fruit Types and Other Morphological Characters

**Fig. 6:**
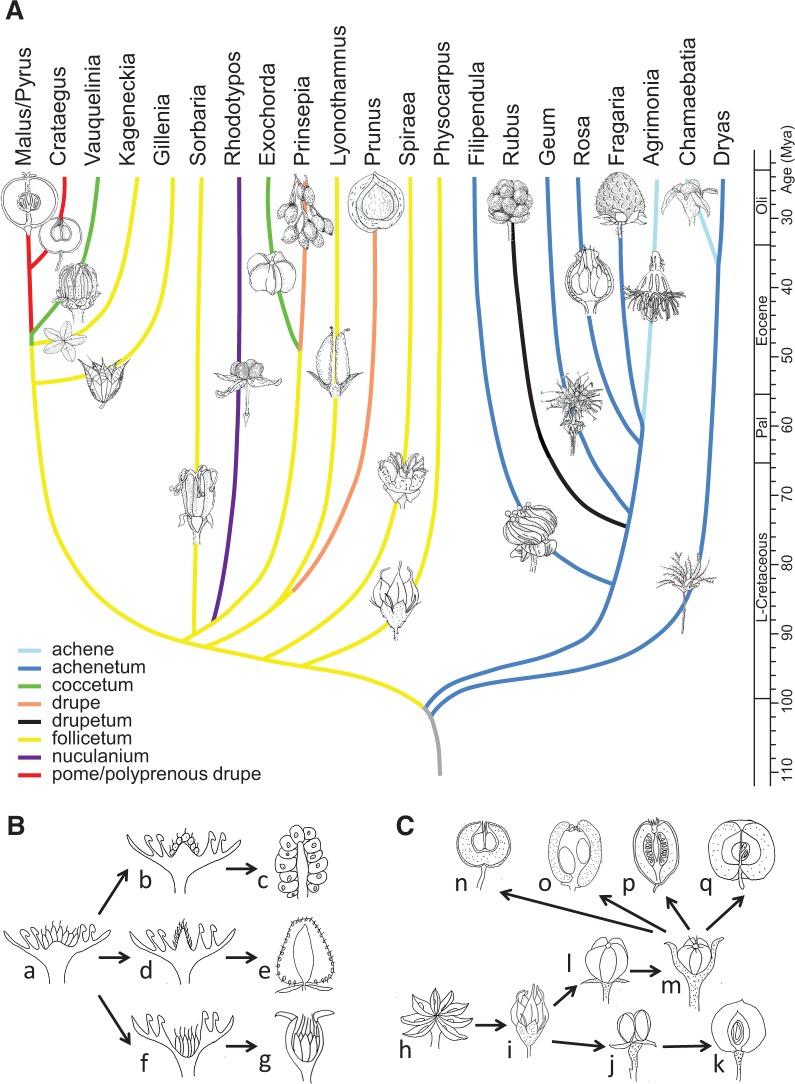
Evolutionary histories of fruit types in Rosaceae. (*A*) The evolutionary history of different fruit types in Rosaceae in the context of an abbreviated phylogeny from than shown in figure 1. Grey line represents ancestral fruit type of Rosaceae. Other line colors represent different fruit types of extant taxa. The positions of nodes and branches correspond with geological time scale on the right redrawn from the molecular clock analysis shown in figure 4. The positions of fruit drawings do not represent the history or age of the fruit types. (*B*) Proposed histories of three fruit types in Rosoideae. a: a hypothetical ancestral fruit of Rosoideae (achenetum); b: a hypothetical ancestral fruit of *Rubus* (drupetum, with less flesh); c: *Rubus* (drupetum, with more flesh); d: a hypothetical ancestral fruit of *Fragaria* (achenetum, without enlargement of receptacle); e: *Fragaria* (achenetum, with fleshy enlargement of receptacle); f: a hypothetical ancestral fruit of *Rosa* (achenetum, with partial enclosure of fruits by the hypanthium); g: *Rosa* (achenetum, with full enclosure by the hypanthium, and increased fruit size). (*C*) Proposed histories of several fruit types in Amygdaloideae. h, i: Hypothetical ancestral fruits of Amygdaloideae, evolving from a follicetum with many (indefinite) carpels (h) to that with five or fewer carpels (i). j: A hypothetical ancestor of Amygdaleae (nuculanium, with a few carpels). k: *Prunus* (drupe, with a single carpel). l, m: hypothetical ancestral fruits of Pyrinae. l evolved into m via the partial “sinking” of the ovary into the hypanthium and fusing with it. n: *Crataegus* (with the endocarp still near the top). o: *Eriobotrya* (with relatively thin flesh). p: *Pseudocydonia* (with many ovules for a carpel, a likely ancestral character). q: *Malus* (with centrally located endocarp along the vertical axis, thick flesh, and one or two seeds per carpel, all likely derived features).

The well-resolved Rosaceae phylogeny provided an excellent opportunity to reconstruct ancestral states of fruit types and other morphological characters. Rosaceae members produce dry fruits, fleshy fruits, or aggregate fruits (Potter et al. 2007a) (fig. 6, supplementary fig. S15, Supplementary Material online). Dry fruits include achene, which has a thin fruit wall with a single seed; some species produce several to many achenes from a single flower, or achenetum (aggregate achenes). Another dry fruit is follicle, which produce one or more seeds within a thin fruit wall that split open at maturity to release the seeds. Again, multiple separate follicle-like fruits within a single flower are referred to as follicetum. Fleshy fruits include drupe, with a thick fleshy fruit wall enveloping a hard shell-like inner fruit wall that surrounds a single seed, and pome, which has thick fleshy accessory tissue (largely derived from the hypanthium, a cup-shaped fusion of lower portions of sepals, petals, and stamens) outside a relatively soft fruit wall containing several seeds. In Rosaceae pomes, the central core is often divided into five small chambers, each with one or a few seeds.

In Dryadoideae, achenes or achenetum (such as in *Dryas*) are the main fruit types. Ancestral character reconstruction supports that achenetum with an enlarged and hemispheric hypanthium, as well as with a very large number of separate carpels in a flower, is the ancestral fruit type of this subfamily (fig. 6*A*, supplementary fig. S15, Supplementary Material online), consistent with the fruit character reconstruction in Potter et al. (2007a) and *Dryas* being the basal lineage of the subfamily. Rosoideae also include species with dry fruits such as some achenetum (Ulmarieae, Colurieae, Roseae, and Potentilleae) and achenes (*Potaninia* and Agrimonieae), as well as fleshy drupetum, or aggregate small drupes (Rubeae). Achenetum with a flat hypanthium such as that in *Filipendula* is the ancestral character, and it seemed to have evolved into three different complex fruit types (fig. 6*B*). In one type, the receptacle becomes somewhat enlarged and hemispherical (such as Potentilleae and Colurieae), and then larger and fleshy with many achenes, such as strawberry (*Fragaria*) and *Duchesnea*. In the second type, the hypanthium extends upward to become urceolate (urn-like) with multiple achenes enclosed within, such as that in *Rosa*. In the third type, the wall of each fruit changes from dry and thin to fleshy and thickened, so that the aggregate fruit has evolved into a drupetum (of multiple small drupelets), such as in raspberry and blackberry (*Rubus*).

In Amygdaloideae, members of several tribes produce the fruit type follicetum (Lyonothamneae, Neillieae, Sorbarieae, Gillenieae, and *Kageneckia* in Maleae), whereas others have one of several distinct types: achenetum (*Neviusia* and *Holodiscus*), coccetum (*Exochorda* and *Vauquelinia*), pome (many genera in Maleae), nuculanium (most of genera in *Kerrieae*), and drupe (Amygdaleae) (fig. 6A, supplementary fig. S15, Supplementary Material online). The ancestral fruits of the subfamily were likely pentamerous aggregate follicles (follicetum), which evolved from an earlier type with an indefinite and larger number of carpels; subsequently, there have been independent further reduction of carpel number for some tribes (such as Amygdaleae and Lyonothamneae). Enlarged and fleshy fruits likely evolved via two distinct ways (fig. 6*C*). In one, the endocarp (inner fruit wall) became hard, forming nuculanium; in addition, the previously dry pericarp (middle fruit wall) became fleshy and the number of carpels decreased to one or two, finally forming the drupe (*Prunus*: peach, plum, cherry, and apricot; *Prinsepia*). Alternatively, after connation of five carpels as coccetum (such as *Vauquelinia)*, the hypanthium become urceolate and fleshy and further closed up with the carpels, evolving into partially inferior (such as *Crataegus*) or fully inferior (such as apple, *Malus*) ovaries. It is worth noting that the newly revolved phylogeny of the subfamily places Amygdaleae with single-carpel fruits as sister to *Lyonothamnus*, which produces follicetum fruits with two separate carpels, suggesting that the reduction of carpel number might be a gradual process.

We have also examined the ancestral states of other characters (supplementary figs. S16–S23, Supplementary Material online). The habit of Rosaceae species most probably evolved from shrubs (supplementary fig. S16, Supplementary Material online). Most Rosoideae species tend to diminish their sizes to perennial or annual herbs with small compound leaves (supplementary fig. S17, Supplementary Material online) and a mass of small dry seeds. On the contrary, trees originated independently in Maleae and Amygdaleae in the subfamily Amygdaloideae, becoming much larger in size with numerous photosynthetic leaves that produce nutrients to support the production of many fleshy fruits. These trees are important members of forests, providing habitats of birds and other animals, which gather fruits and seeds, sometimes for future use in locations away from the plants, thereby facilitating the spread of seeds. In addition to changes in size and number of leaves, compound leaves also evolved a few times independently within Amydaloideae (supplementary fig. S17, Supplementary Material online).

As for flowers, most Rosaceae species share some common ancestral characters (supplementary figs. S18–S23, Supplementary Material online), such as bearing a hypanthium, which is a connation of the receptacle and the basal part of perianth and stamens, and having pentamerous sepals and petals (wind-spread species tend to have no petals). However, pistils are distinctive in different clades. An apocarpous pistil with superior ovary and numerous carpels is the ancestral character of Rosaceae. Most Rosoideae species have maintained this character, while carpel number tends to diminish to five (such as most species in Amygdaloideae) and even to one (such as species in Amygdaleae and some species in Dryadoideae), allowing greater supply of nutrients to each fruit. Furthermore, Maleae species produce inferior pistil, probably ensuring better protection for ovules.

### Possible Effects of WGD and Climate Changes on Fruit Evolution

The nuclear phylogenetic analyses here produced a robust Rosaceae phylogeny, which has served as a framework for further analyses, including molecular clock estimates of ages of Rosaceae lineages and genome-scale analyses of gene family evolution with strong evidence for multiple WGDs. Furthermore, ancestral character reconstruction for fruit types support independent origins of several fleshy fruit types from ancestral dry fruits, including different forms of pomes in many genera of Maleae, drupes in *Prunus* species (peach, plum, cherry, and apricot), nuculanium with 1–5 small fruits in many species in Kerrieae, and drupetum with multiple drupelets in *Rubus* (such as raspberry and blackberry). In addition, strawberry has evolved a complex fruit structure with a fleshy hypanthium with many achenes attached to its surface. The evolution of fleshiness includes the enlargements of pericarp (drupe, drupetum, or nuculanium), hypanthium/receptacle (strawberry-like), or both (pome).

The fleshy fruits in the subfamily Amygdaloideae are concentrated in the tribes Maleae and Amygdaleae, both of which also have evolved trees, whereas the aggregate fruits with fleshy tissues in Rosoideae are from tribes with herbaceous (Potentilleae; *Fragaria*) or bushy (Rubeae; *Rubus*) habits. The Maleae and Amygdaleae lineages with fleshy fruits exhibit strong evidence of having had lineage-specific WGDs because of the divergence times of these tribes from others. In particular, the large and diverse tribe Maleae probably had two WGDs, one shared by all genera examined here and was likely in the most recent common ancestor (MRCA) of the tribe, and the other was shared by all fleshy-fruit bearing members included here, and likely occurred in the MRCA of the subtribe Pyrinae. The successive WGDs might have provided many more genes to allow for functional innovations after the duplications via dosage effects, neofunctionalization, or subfunctionalization (Blanc and Wolfe 2004; Pontes et al. 2004; Madlung et al. 2005; Cusack and Wolfe 2007; Freeling 2009; Bekaert et al. 2011; Hudson et al. 2011). This is corroborated by the evolution of multiple sub-types of pomes in Maleae, with an evolutionary trajectory from superior to inferior ovaries, from five distinct carpels to fused carpels, from thin and nonfleshy hypanthium/pericarp to fleshy tissues, and possibly from multiple ovules to few ovules attached to each carpel (fig. 6).

With the support of highly resolved relationships between genera (figs. 1 and 6), we propose the following scenario for fruit evolution in Maleae: (1) the ancestor produced follicetum fruits with five carpels, as also seen in the basal lineage *Kageneckia*; (2) in the next basal clade, *Crataegus* and related genera share a form that has a semi-inferior ovary separated into five parts; this might be an intermediate form of fleshy fruit during evolution; (3) in the next clade, *Eriobotrya* has a fruit with a fleshy tissue being thinner at the top, suggesting that the enclosure of the ovary by the cup-shaped hypanthium is incomplete; (4) in the clade with *Cydonia, Chaenomeles*, and *Pseudocydonia*, these three genera produce fruits with several ovules for each carpel, suggesting some degree of primitiveness; and (5) the fruits of *Sorbus*, *Pyrus*, and *Malus* all have inferior ovaries, suggesting more derived forms.

In the tribe Maleae, the timing of fruit character transitions is closely correlated with those of WGDs and climate events, suggesting possible impacts of WGD and climate factors on fruit evolution. Molecular clock estimates suggest that the tribe Maleae split from Gillenieae at around 54 Ma just after the Paleocene–Eocene boundary, with further divergences within Maleae beginning shortly afterwards. We found evidence for two closely spaced WGDs near the origin of Maleae. The earlier WGD shared by all Maleae members occurred at early Eocene, which was the hottest period in the Cenozoic Era, including both the Paleocene–Eocene Thermal Maximum (PETM) and Early Eocene Climate Optimum (EECO) (Zachos et al. 2008). Within Maleae, after the separation of *Kageneckia* (with fruit type of follicetum), the ancestor of *Vauquelinia* and other genera likely produced the fruit type coccetum, with a short lag period from the early WGD event. The second WGD was shared by the fleshy-fruited genera of Maleae (fig. 5) and occurred in late Eocene (fig. 4), when the Earth experienced continuous decrease in temperature and humidity, closely followed by a short glaciation period with extinctions of many species in Europe (Zachos et al. 2001; Hooker et al. 2004). The unusually high percentage (50.12%) of retained gene pairs of the WGD and the rapid taxon separation/diversification after the WGD suggest that the duplicate genes from the WGD contributed to the diversification of the decent Maleae genera. Thus it is possible that the new gene copies from two Maleae WGDs allowed the descendant Maleae members to evolved new fruit types under the selective forces of both the dramatic climate changes from early to late Eocene and the interactions with animals that lived in the forests of woody Maleae members and ate their fleshy fruits.

The potential for new gene functions offered by the WGDs might also have contributed to the evolution of relatively large trees in Maleae and Amygdaleae, as compared with herbs and bushes in other Rosaceae tribes. The tree habit that can reach greater heights for light exposure and that have more leaves to harvest the light energy could also give these tribes greater advantages, by allowing them to produce many more fruits per plant, and more fleshy fruits to attract animals. In contrast, the fleshy fruits of *Rubus* and *Fragaria* seems to have evolved via different paths, and are not associated with obvious lineage-specific WGDs or tree habit. Human efforts have resulted in the domestication of several fleshy-fruited Rosaceae species, increasing the sizes and sweetness of the fruits. Still other Rosaceae tribes/genera have retained the earlier dry fruit types of achene and follicle (or related achenetum and follicetum, respectively), yet some of them have developed appendages to facilitate attachment to animals (such as hooks in *Geum*) or spread by wind (*Dryas*). The multiple independent origins of distinct fleshy fruits discussed in this study suggest a general trend of fleshy fruit evolution, including the decrease of carpel number, the extension of hypanthium/receptacle to enclose and, sometimes, fuse with the ovary, as well as the enlargement and softening of ovary wall, hypanthium, or receptacle. Such scenario for the evolution of fleshy fruits might also exist in other families, especially those with both dry and fleshy fruits, such as citrus, melons, and tomatoes (Gu et al. 2003). This study provides a foundation combining molecular phylogeny with molecular clock estimates and evidence for WGDs, placing the evolution of Rosaceae fruit types in the context of geological ages and related climate changes, as well as genome-scale changes that allow potential functional innovation. The wide range of fruit types, available genomic resources of Rosaceae species and model system for functional analyses, and the phylogenetic framework presented here all contribute to making Rosaceae an excellent system for studying fruit evolution, an important problem for understanding angiosperm evolution and improving fruit crops for horticulture.

## Materials and methods

### Tissue Collection, RNA Isolation, Transcriptome Sequencing, and Sequence Retrieval

Young leaves, buds or fruits were collected and frozen at −80 °C. RNA isolation, cDNA synthesis, high-throughput sequencing, sequence assembly were carried out as previously described (Huang et al. 2016). Genomes and Sequence Read Archive data sets were retrieved from Phytozome (https://phytozome.jgi.doe.gov/pz/portal.html; last accessed November 12, 2016), Mei Genome Project website (http://prunusmumegenome.bjfu.edu.cn/; last accessed June 13, 2016), Pear Genome Project website (http://peargenome.njau.edu.cn/; last accessed November 12, 2016), and GenBank (http://www.ncbi.nlm.nih.gov/genbank/; last accessed November 12, 2016).

### Identification of Low Copy Candidate Orthologous Genes

In the following investigations, sequence retrieval from public or in-house datasets was performed using HaMStR v13.2.3 (Ebersberger et al. 2009) with *E* value of less than e^−^^20^. Maximum likelihood (ML) phylogenetic analysis for single-gene families was performed using RAxML v7.0.4 (Stamatakis 2006) with 100 bootstrap replicates under GTRGAMMAI model as recommended by jModelTest v2.1 (Darriba et al. 2012) (see next section for more on phylogenetic analysis). The scripts for the bioinformatics analyses here are available if requested.

For use as phylogenetic markers, low copy nuclear genes were identified from two groups of genes (supplementary fig. S24A, Supplementary Material online). The first group contains 931 candidate orthologous genes (Zeng et al. 2014) in an overlapping set of orthogroups between two datasets (using the *Arabidopsis thaliana* gene ID numbers present in both sets): one includes 4180 orthologous genes shared by nine angiosperm genomes (*Arabidopsis thaliana, Populus trichocarpa, Glycine max, Medicago truncatula, Vitis vinifera, Solanum lycopersicum, Oryza sativa, Sorghum bicolor*, and *Zea mays*) retrieved from the Deep Metazoan Phylogeny (http://www.deep-phylogeny.org/hamstr/; last accessed November 12, 2016) website, the other includes 1989 orthologous genes obtained from seven genomes (*Arabidopsis thaliana, Populus trichocarpa, Prunus persica, Vitis vinifera, Mimulus guttatus, Oryza sativa*, and *Sorghum bicolor*) using OrthoMCL (Li et al. 2003). Using the 931 seeds generated in Zeng et al. (2014) and HaMStR with *E* value of less than e^−^^20^, we obtained homologs of these 931 genes from eight representative Rosaceae species (*Fragaria vesca, Rubus coreanus, Crataegus pinnatifida, Malus × domestica, Spiraea japonica, Prunus dulcis, Prunus persica*, and *Prunus mume*) and two outgroup species (*Medicago truncatula* and *Cucumis sativus*), which have well-established relationships. Among these, 546 genes were retained that had length of 1000 base pairs or more in each of four Rosaceae genomes (*Fragaria vesca, Malus × domestica, Prunus persica*, and *Prunus mume*) and *Medicago truncatula*, as an outgroup, and coverage of more than 60% of the above mentioned 10 species (8 Rosaceae and 2 outgroups) using in-house scripts. To avoid hidden paralogs, single-gene trees of the 546 OGs from the ten species were reconstructed using RAxML then inspected manually and 407 genes whose tree topologies did not contradict with the organismal tree (supplementary fig. S24B, Supplementary Material online) of the 10 species were retained.

In addition, 3863 single copy nuclear genes that were shared by four species (*Fragaria vesca, Prunus Persica, Prunus mume*, and *Cucumis sativus*) were identified by using MCScan v0.8 (Wang et al. 2012). To avoid selecting the same genes, the 3863 orthologs were identified in *Arabidopsis thaliana*, and by comparing gene IDs, those overlapping with the above-mentioned 4180 gene set were excluded. Then 2124 genes with sequence length of 1000 base pairs or more in *Cucumis sativus* were selected using in-house scripts. Among the 2124 genes, those with only one or two copies in *Malus × domestica* and *Pyrus bretschneideri* were retained, resulting in 475 genes.

The combined set (407 + 475) of 882 genes was retrieved from 124 Rosaceae species and 24 outgroup species using 407 seeds from Zeng et al. and 475 seeds generated in this study respectively by HaMStR, and was used to construct single-gene family trees by RAxML. The tree topologies were examined manually for inconsistencies with well-established known relationships; 311 genes that grouped members of different orders together were removed (resulting in 571 genes), then 127 genes failing to group members of Rosaceae subfamily were removed (yielding 444 genes), then 188 genes with conflict for monophyly of tribes were eliminated (with 256 remaining). Among the relationships in Maleae, four clades of two or three species received maximum support from all of the 882, 571, 444, and 256 gene sets: (*Malus *×* domestica*, *Malus baccata*), (*Eriobotrya japonica*, *Rhaphiolepis indica*), (*Crataegus cuneata*, *Mespilus germanica*), and (*Photinia villosa*, *Sorbus keissleri*, *Stranvaesia amphidoxa*). Among the 256 single-gene trees, 113 genes yielded single-gene trees with these four clades and were remained for further phylogenetic and other molecular evolutionary analyses.

To test whether the recent WGD events in Maleae affected the identification of orthologous genes in Maleae species, a gene set contains 484 genes with only one copy in *Pyrus bretschneideri* were selected from 2124 genes using in-house scripts. Then 484 gene family trees were constructed using RAxML v7.0.4 with homologous sequences retrieved using HaMStR from 31 species including 25 species in Maleae (supplementary table S1, Supplementary Material online) and, as outgroups, *Gillenia stipulata, Gillenia trifoliata, Prunus persica, Prunus mume, Neillia sinensis*, and *Physocarpus opulifolius*. These gene trees were examined manually and treated as follows: (1) if most of the species have only one copy in the single gene tree, even if some species have more than one copy from very recent lineage-specific duplication, this kind of genes were retained and (2) if most of the species have more than one copy, the clade with more species was retained. Finally, 163 genes remained with at most one copy per species.

### Alignment and Phylogenetic Analysis

Phylogenetic reconstruction was performed with all gene sets using coalescence method implemented in ASTRAL v4.4.4 (Mirarab et al. 2014). A maximum likelihood (ML) analysis was also performed with the 113 gene sequence supermatrix using RAxML v7.0.4 (Stamatakis 2006) under GTRGAMMAI model as recommended by jModelTest v2.1 (Darriba et al. 2012). 882 orthologous genes from 148 species were identified using HaMStR. Nucleotide sequences of each orthologous genes were aligned using MUSCLE v3.8.31 (Edgar 2004) with default parameters. Then the regions poorly aligned were further trimmed using trimAl v1.2 (Capella-Gutiérrez et al. 2009). Single-gene trees were reconstructed using RAxML v7.0.4 (Stamatakis 2006) under GTRCAT model. For each gene groups, 100 bootstrap replicates were generated respectively for the coalescent analysis (Mirarab et al. 2014). The nucleotide sequence alignments of 882 orthologous genes in 148 species are accessible in TreeBASE website (http://treebase.org/treebase-web/home.html; last accessed November 12, 2016), with the submission number of 19726.

### AU Test

CONSEL v0.1j (Shimodaira and Hasegawa 2001) was used to test the alternative topologies in two phases as previously described in Huang et al. (2016).

### Molecular Clock Estimation of Geological Ages

Divergence times were estimated using the 113-gene ML tree and 19 fossil constraints (information for all fossils and their assignments can be found in supplementary table S3, Supplementary Material online), with the penalized likelihood (PL) method implemented in r8s (Sanderson 2003) and the optimum smoothing value of 0.01 according to the build-in cross validation procedure to correct rate heterogeneity among lineages. The r8s method was used instead of BEAST because the former has been shown to be effective in recent studies (Carbonell-Caballero et al. 2015; de Casas et al. 2016; Feng et al. 2016; Plazzi et al. 2016) and the latter would require much more computational time with this large datasets here. The fossils used here were from a survey of literature and online resources on Paleobiology Database website (https://www.paleobiodb.org/; last accessed November 12, 2016) for the oldest fossil of the corresponding MRCA nodes. Among the 19 fossil constraints, 13 were for Rosaceae, described as follows (numbers correspond to those in fig. 4): (1) Fossils of *Amelanchier peritula* and *Amelanchier scudderi* were discovered in the Florissant Formation, Colorado, USA, and have been dated to Chadronian in Late Eocene (37.2–33.9 Ma) (Cockerell 1911; MacGinitie 1953). We assigned the fossils to stem *Amelanchier* with a minimum age of 33.9 Ma. (2) Fossil *Vauquelinia comptonifolia* found in the Green River Formation in Wyoming, USA, has been dated to 46.2–40.4 Ma in Uintan, Middle Eocene (MacGinitie 1969). Thus, we constrained stem *Vauquelinia* with a minimum age of 40.4 Ma. (3 and 7) Fossils of *Neviusia* and *Spiraea* sp. found in Republic, Washington, USA have been dated to 49–50 Ma in Early Eocene (Mathews 1964; Wehr and Hopkins 1994). Both of them were assigned to the stem node correspondingly with minimum age of 48.6 Ma corresponding to the upper boundary of Early Eocene. (4) Fossils of *Oemleria janhartfordae* were uncovered in the Klondike Mountain Formation, Washington, USA and were dated to 49.42 ± 0.54 Ma in Early Eocene (Wolfe et al. 2003; Greenwood et al. 2005; DeVore and Pigg 2007; Benedict et al. 2011). The minimum age of stem *Oemleria* was constrained to 48.6 Ma corresponding to the upper boundary of Early Eocene. (5) Fossil *Prunus wutuensis* found in the Wutu Formation, Shandong Province, China, has been dated to 55 Ma in Early Eocene (Li et al. 2011). We used this age (55 Ma) to constrain stem *Prunus*. (6) *Holodiscus lisii* found in Florissant, Colorado, USA, has been dated to 34.1 Ma in Late Eocene (Schorn 1998; McIntosh and Chapin 2004). We set the minimum age of stem *Holodiscus* to 34.1 Ma for this fossil calibration. (8) Macrofossils of *Fragaria* were discovered in the Beaufort formation, Prince Patrick Island in the Canadian Arctic, and the age was considered to be about 2.96 Ma in Late Pliocene, corresponding to the age of Lost Chicken tephra in Alaska (Matthews and Ovenden 1990; Matthews et al. 2003). We used this fossil to calibrate the minimum age of stem *Fragaria* to 2.96 Ma. (9) Microfossils of *Acaena* sp. were found in Cullen Formation in Tierra del Fuego, Argentina, which were dated to 48.6–37.2 Ma in Middle Eocene (Zetter et al. 1999). This fossil was used to calibrate stem *Acaena* with 37.2 Ma as a minimum age constraint. (10) Mesofossil of *Rosa germerensis* belongs to the Challis Volcanics Formation in Custer County, Idaho, which has been dated to 55.8–48.6 Ma in Early Eocene (Edelman 1975). We calibrated this fossil to stem *Rosa* with a minimum age of 48.6 Ma. (11) *Rubus acutiformis* was discovered in Dorset, United Kingdom and dated to 47.8–41.3 Ma in Lutetian, Middle Eocene (Chandler 1963). Thus, we constrain the minimum age of stem *Rubus* to 41.3 Ma. (12) Fossil *Cercocarpus myricaefolius* found in the Florissant Formation, Colorado, USA has been dated by a single-crystal ^40^Ar/^39^Ar analysis of sanidine from pumice in sandstone and debris flow deposits, resulted in a mean age of 34.07 Ma in Late Eocene (MacGinitie 1953; Evanoff et al. 2001). This mean age was used here to calibrate the minimum age of stem *Cercocarpus*. (13) The oldest fossil of Rosaceae is a macrofossil found in the Aspen Shale Formation, Wyoming, USA and been dated to 113.0–100.5 Ma in Albian, Early Cretaceous (Peppe et al. 2008). We used this fossil to calibrate the minimum age of stem Rosaceae to 100.5 Ma in our analysis.

### Detecting Whole Genome Duplication by Paralog Gene Trees

We performed all-against-all BLASTP with *e*-value cutoff of 10^−^^5^ among gene sequences of 124 Rosaceae species and 24 outgroups (supplementary table S1, Supplementary Material online) to search for homologous genes, and removed genes showing sequence identity < 50%. These genes were then divided into orthologous groups using OrthoMCL v1.4 (Li et al. 2003) with inflation value of 2.0. Ortho groups (OGs), in addition, taxon coverage of 85% or greater was used to balance between the needs to maximize gene number and minimize missing data, totaling 9482 gene families, were retained for subsequent analyses. Amino acid sequences predicted from genes in each of the OGs were aligned by MUSCLE v3.8.31 (Edgar 2004) with default parameters. These alignments were used for phylogenetic reconstructions using RAxML under the GTRCAT model, and the resulting gene family trees were compared with organismal tree in this study (fig. 1) to map the positions of gene duplications. When a node gaining BP support > 50% and having the same two species in each of its duplicated subclades, a gene duplication was mapped and counted to the corresponding position of the organismal tree. We also required the last common ancestor (LCA) of the subclades to share the same or close depth with the differences smaller than one step, while the steps were determined as the stages traveling from a node to the root in the species tree. The above criteria were implemented literately for all gene family trees, and the numbers of duplicated trees were summarized for each node.

### Ancestral Character Reconstruction

The morphological information of the characters (supplementary table S7, Supplementary Material online) was obtained from the book Flora of North America (floranorthamerica.org), Flora Republicae Popularis Sinicae (http://frps.eflora.cn/; last accessed November 12, 2016), and Global Plants JSTOR (http://plants.jstor.org/; last accessed November 12, 2016). A monophyletic genus was identified as a unit to construct a morphological character matrix. For a genus that is not monophyletic, more than one unit was used to present characters of different clades. The ancestral characters were analyzed using Mesquite v3.04 (Maddison and Maddison 2004) based on the topology in figure 1.

## Supplementary Material

Supplementary data are available at *Molecular Biology and Evolution* online.

## Note Added in Proof

The whole-genome duplication (WGD) analyses performed here used a method developed by Dr Ji Qi at Fudan. During the reviewing process of this paper, a paper describing the WGD analysis was published and should be the reference for the method: Huang C.-H., Zhang, C., Liu, M., Hu, Y., Gao, T., Qi, J.*, Ma, H.* 2016. Multiple paleopolyploidization events across Asteraceae with two nested events in the early history revealed by nuclear phylogenomics. *Mol Biol Evol*. 33(11):2820–2835. (* co-corresponding authors)

## Supplementary Material

Supplementary Data
