# Supplementary material for: Evolution of Rosaceae Fruit Types Based on Nuclear Phylogeny in the Context of Geological Times and Genome Duplication
Source: Mol Biol Evol. 2016 Nov 17;34(2):262–81. doi: 10.1093/molbev/msw242 (PMC5400374; doi:10.1093/molbev/msw242)
Supplement: Supplementary Data [file msw242_Supp.zip › Supplementary-Figures.pdf]

Figure S1

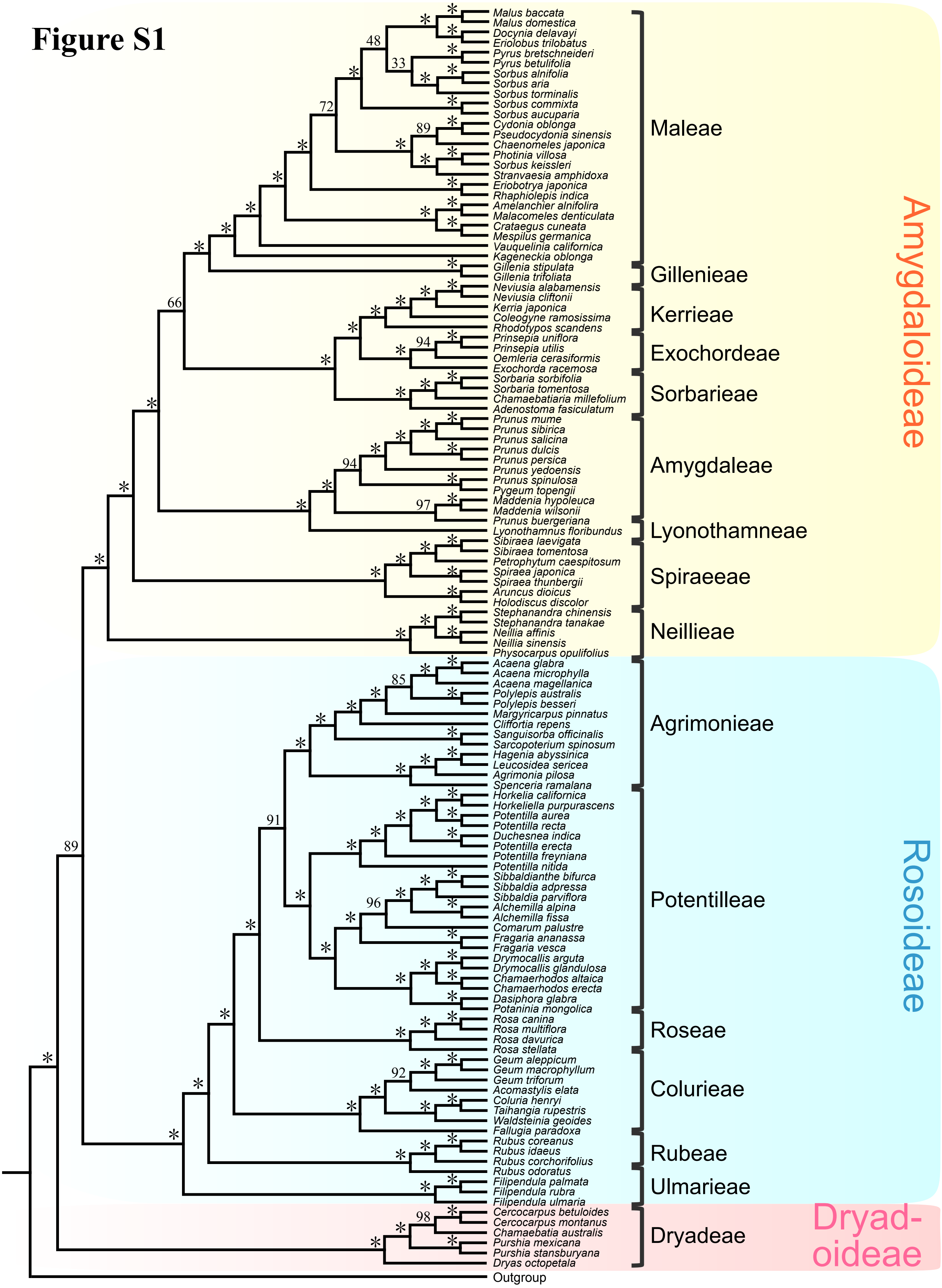

Figure S1. A phylogeny from coalescence analysis using 882 genes.

Numbers indicate values of support obtained by ASTRAL. Asterisks (*) indicate 100% support.

Figure S2

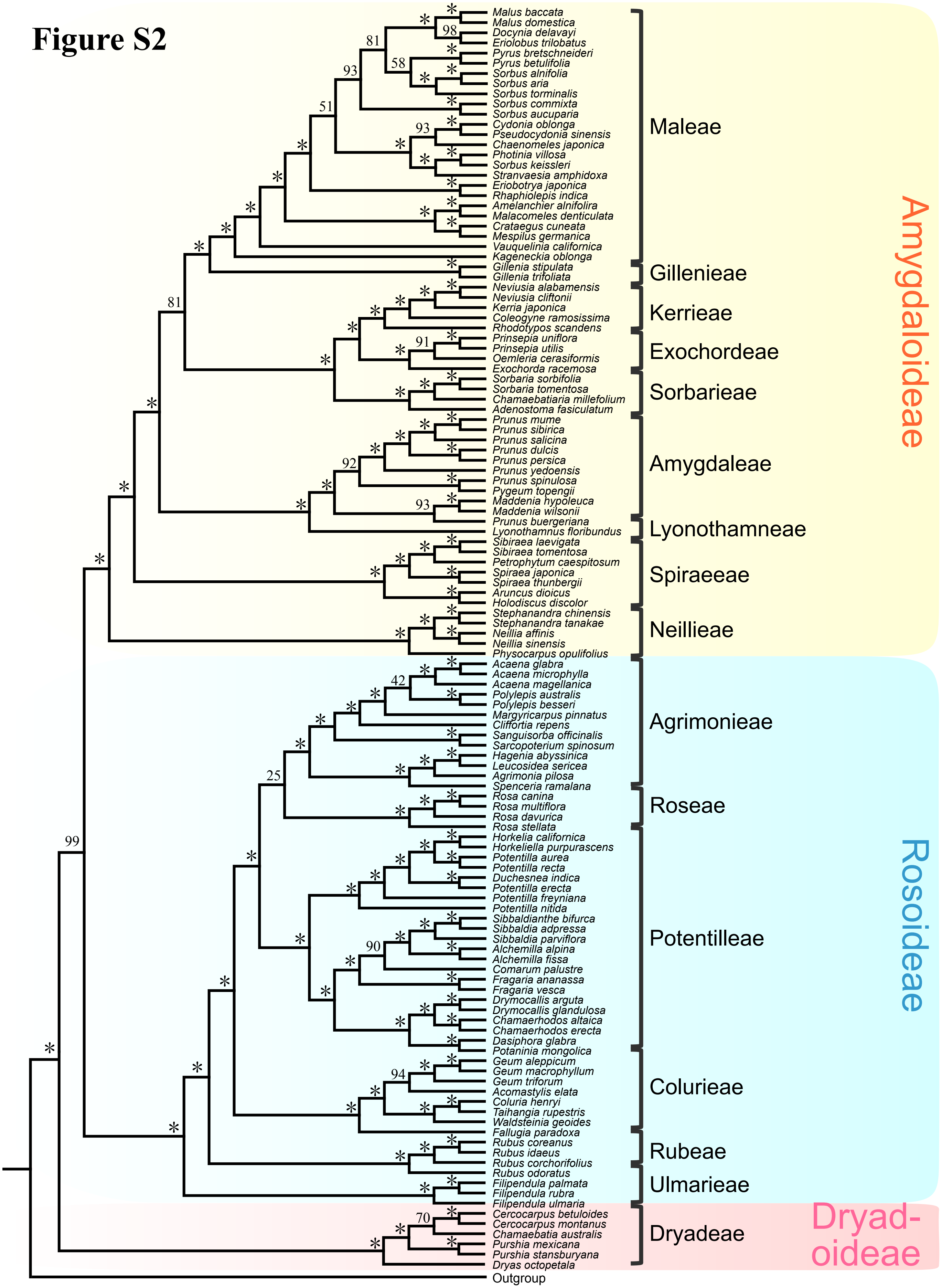

Figure S2. A phylogeny from coalescence analysis using 571 genes.

Numbers indicate values of support obtained by ASTRAL. Asterisks (*) indicate 100% support.

Figure S3

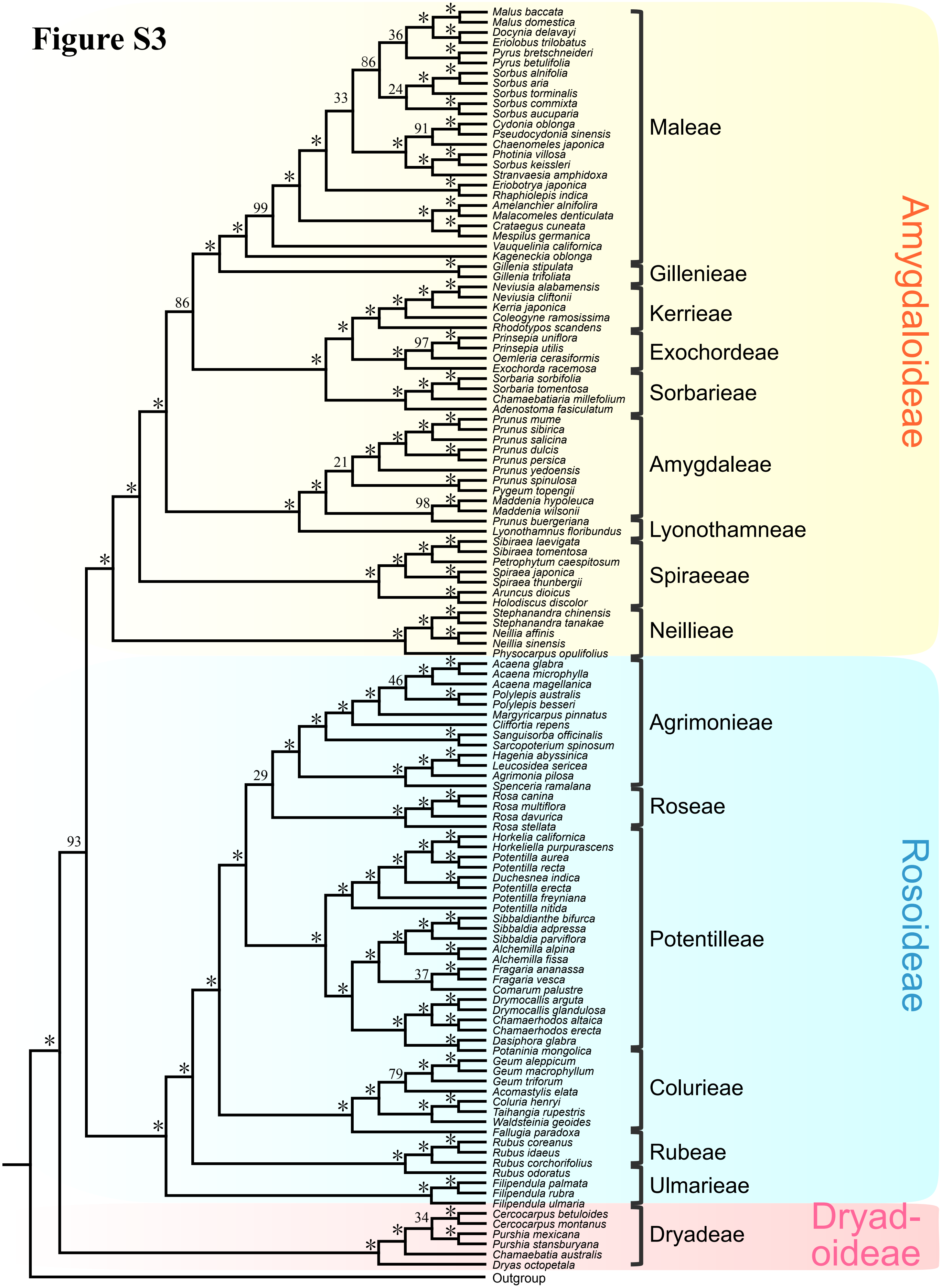

Figure S3. A phylogeny from coalescence analysis using 444 genes.

Numbers indicate values of support obtained by ASTRAL. Asterisks (*) indicate 100% support.

Figure S4

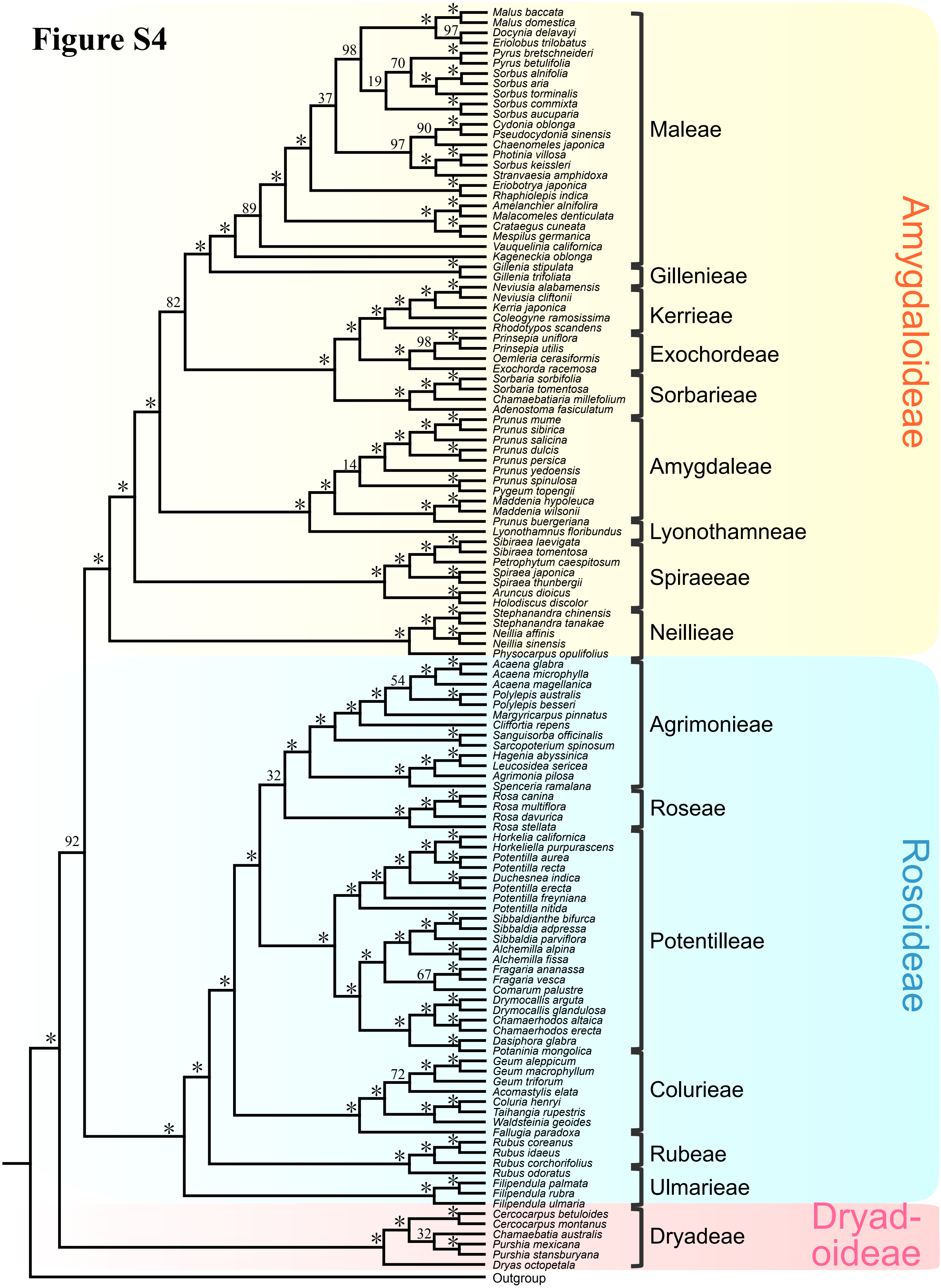

Figure S4. A phylogeny from coalescence analysis using 256 genes.

Numbers indicate values of support obtained by ASTRAL. Asterisks (*) indicate 100% support.

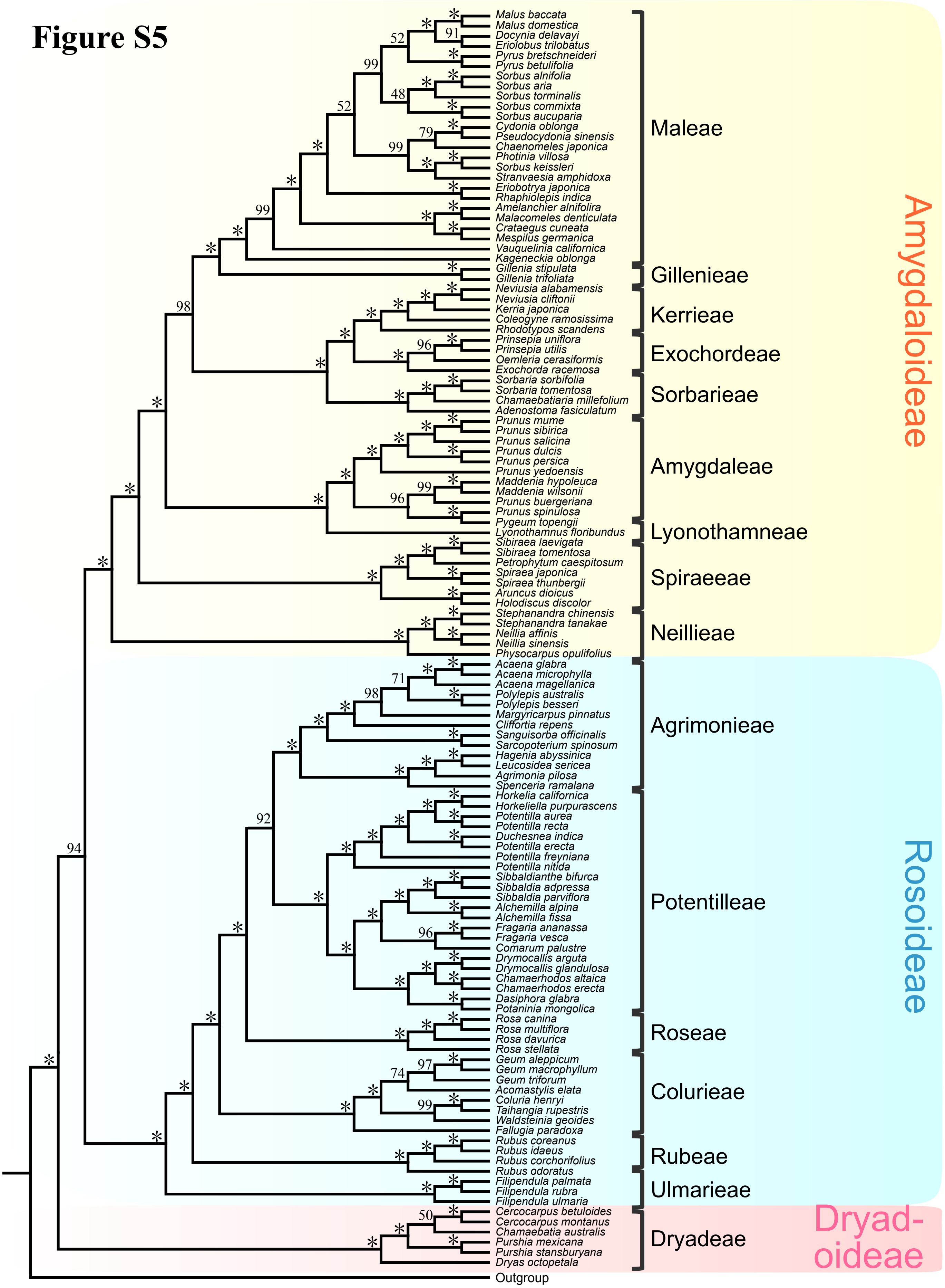

Figure S5. A phylogeny from coalescence analysis using 113 genes.

Numbers indicate values of support obtained by ASTRAL. Asterisks (*) indicate 100% support.

Figure S6

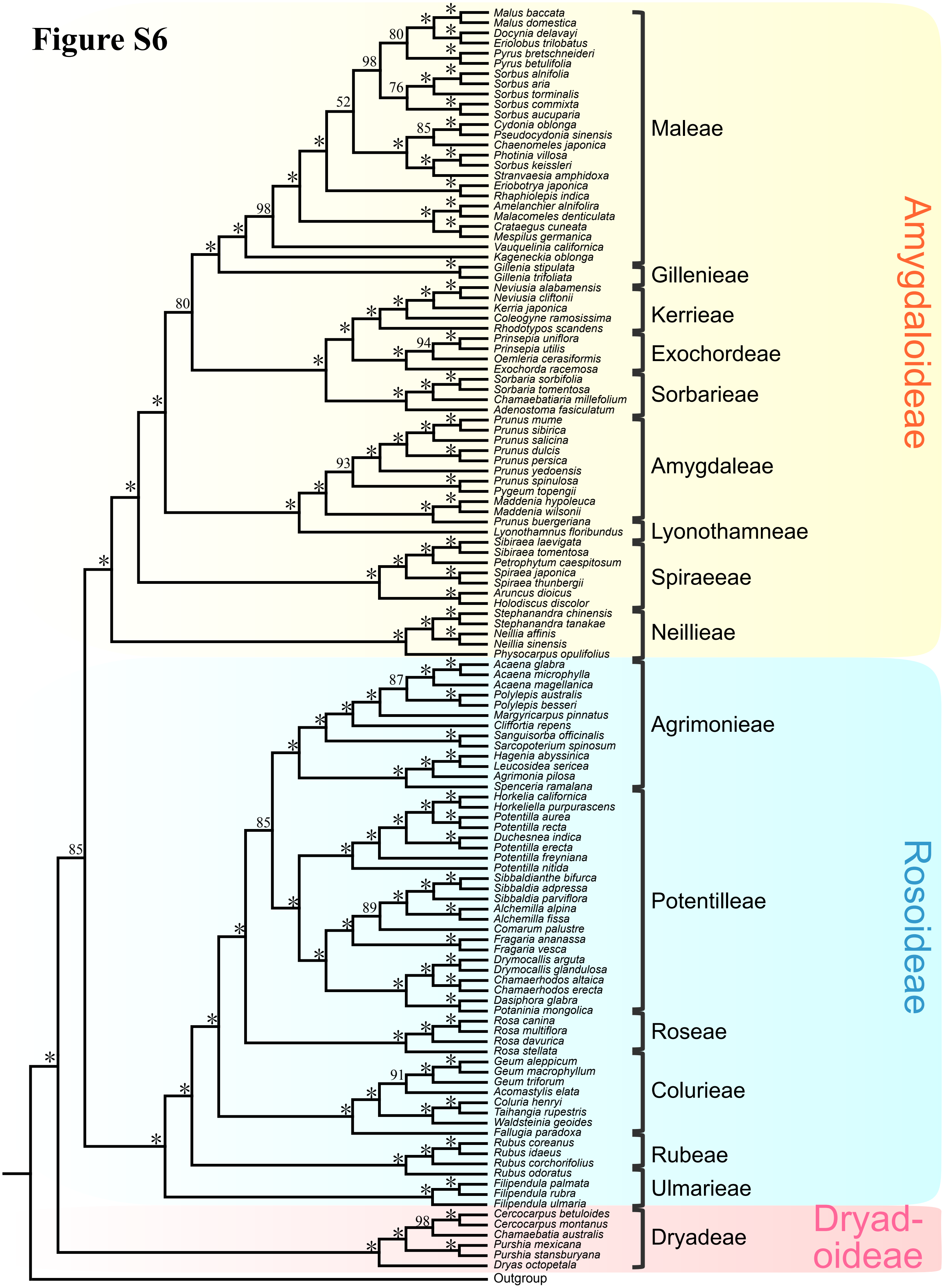

Figure S6. A phylogeny from coalescence analysis using 797 genes.

Numbers indicate values of support obtained by ASTRAL. Asterisks (*) indicate 100% support.

Figure S7

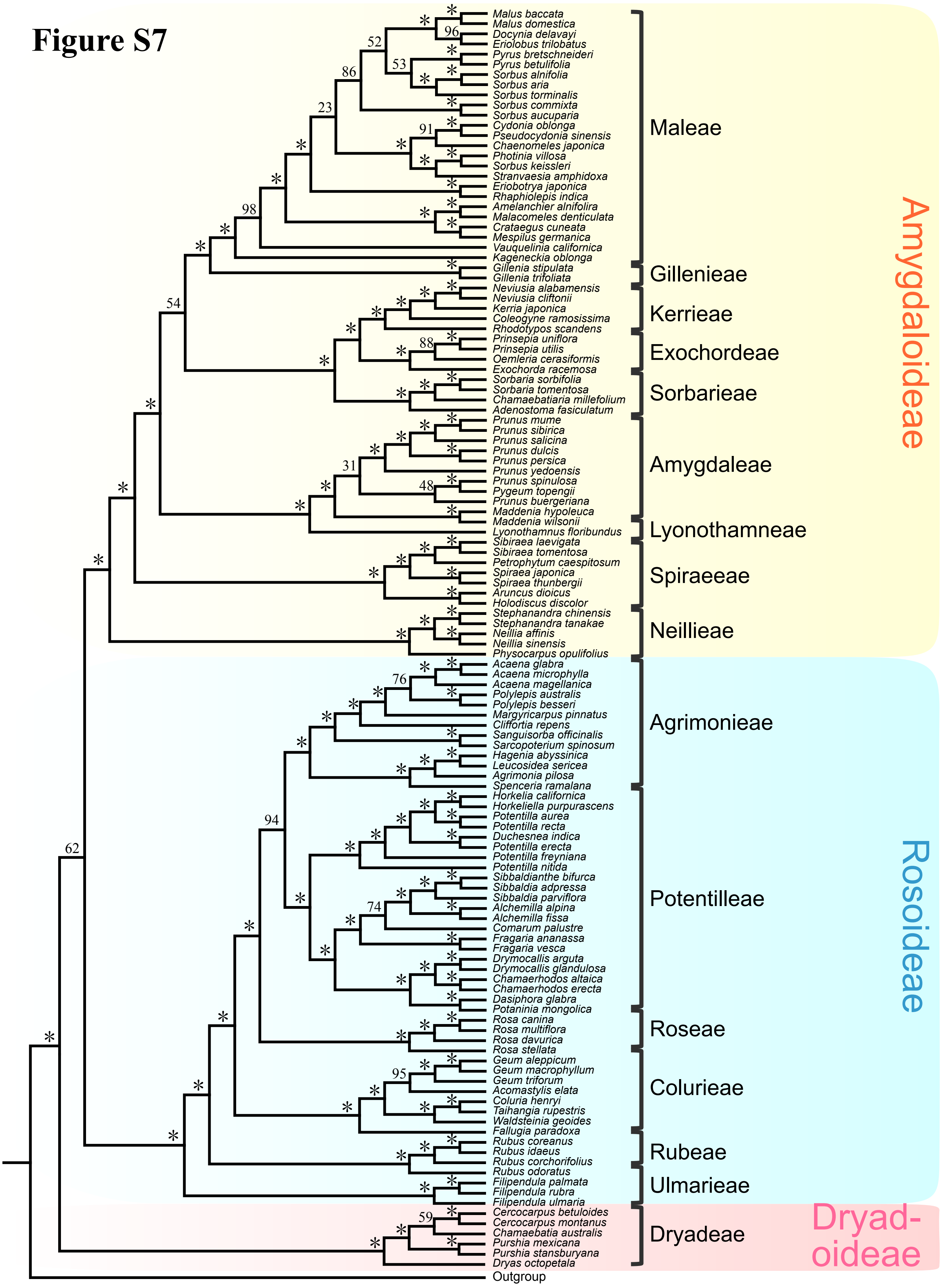

Figure S7. A phylogeny from coalescence analysis using 407 genes.

Numbers indicate values of support obtained by ASTRAL. Asterisks (*) indicate 100% support.

Figure S8

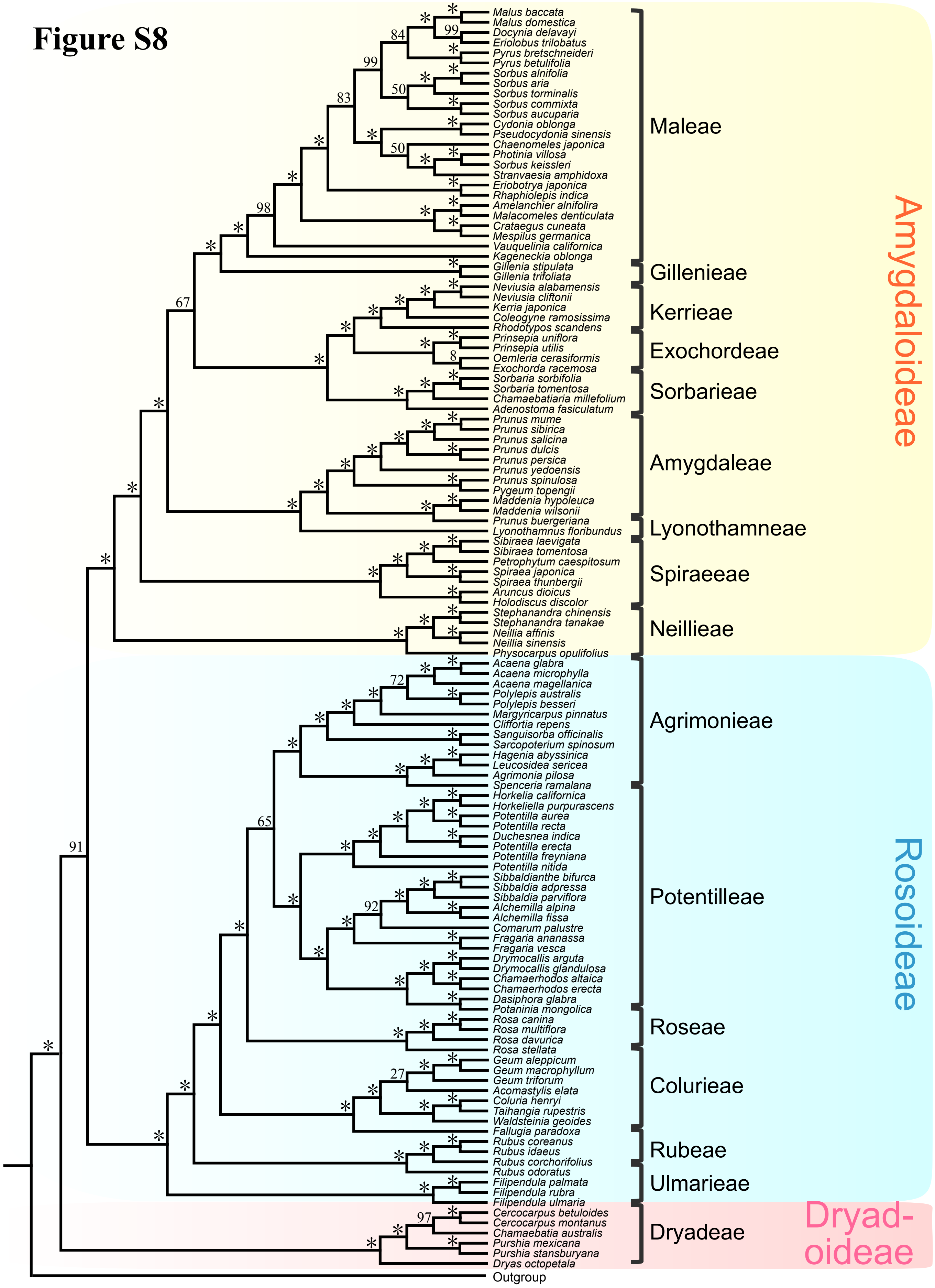

Figure S8. A phylogeny from coalescence analysis using 475 genes.

Numbers indicate values of support obtained by ASTRAL. Asterisks (*) indicate 100% support.

Figure S9

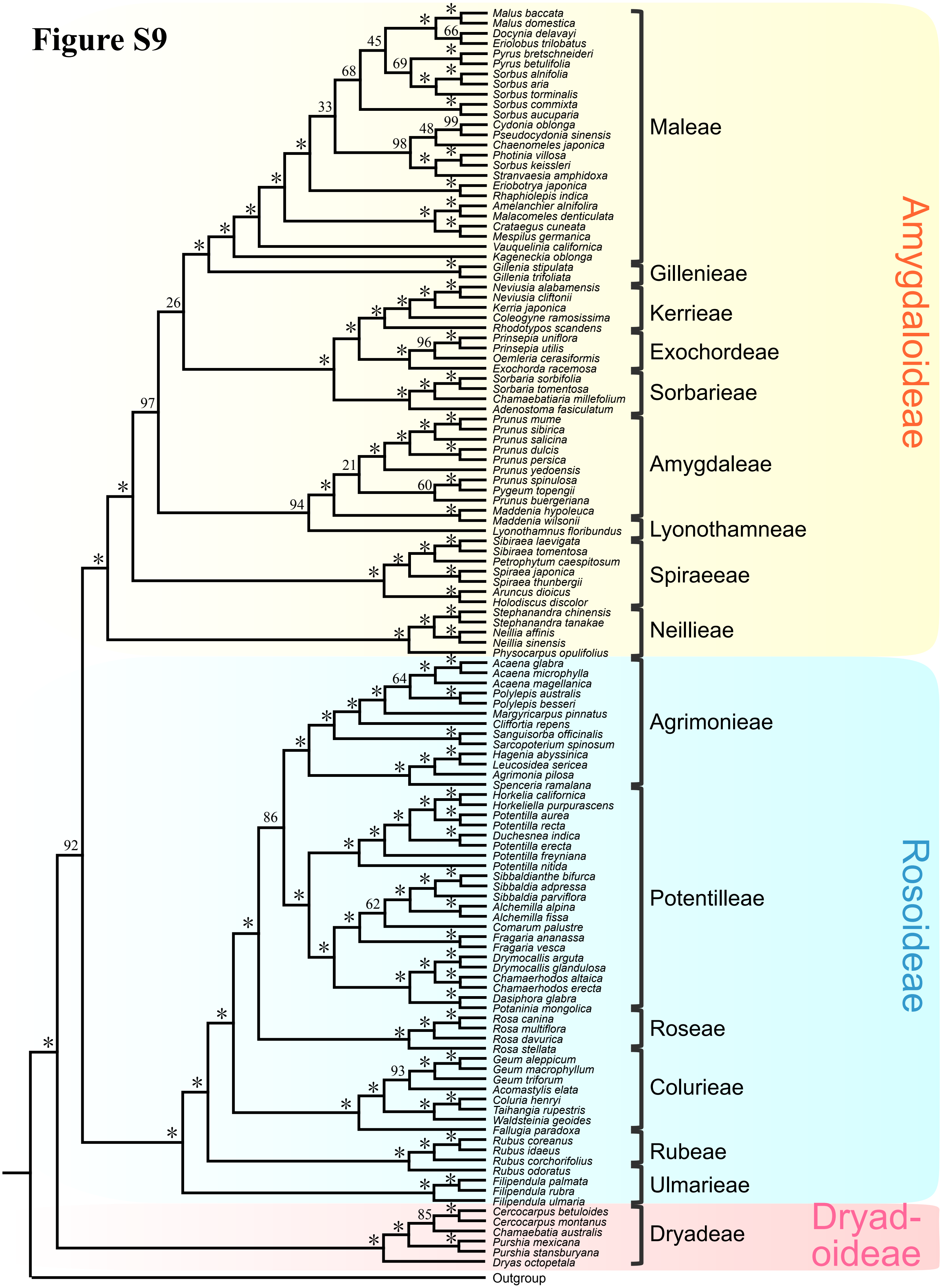

Figure S9. A phylogeny from coalescence analysis using 241 genes.

Numbers indicate values of support obtained by ASTRAL. Asterisks (*) indicate 100% support.

Figure S10

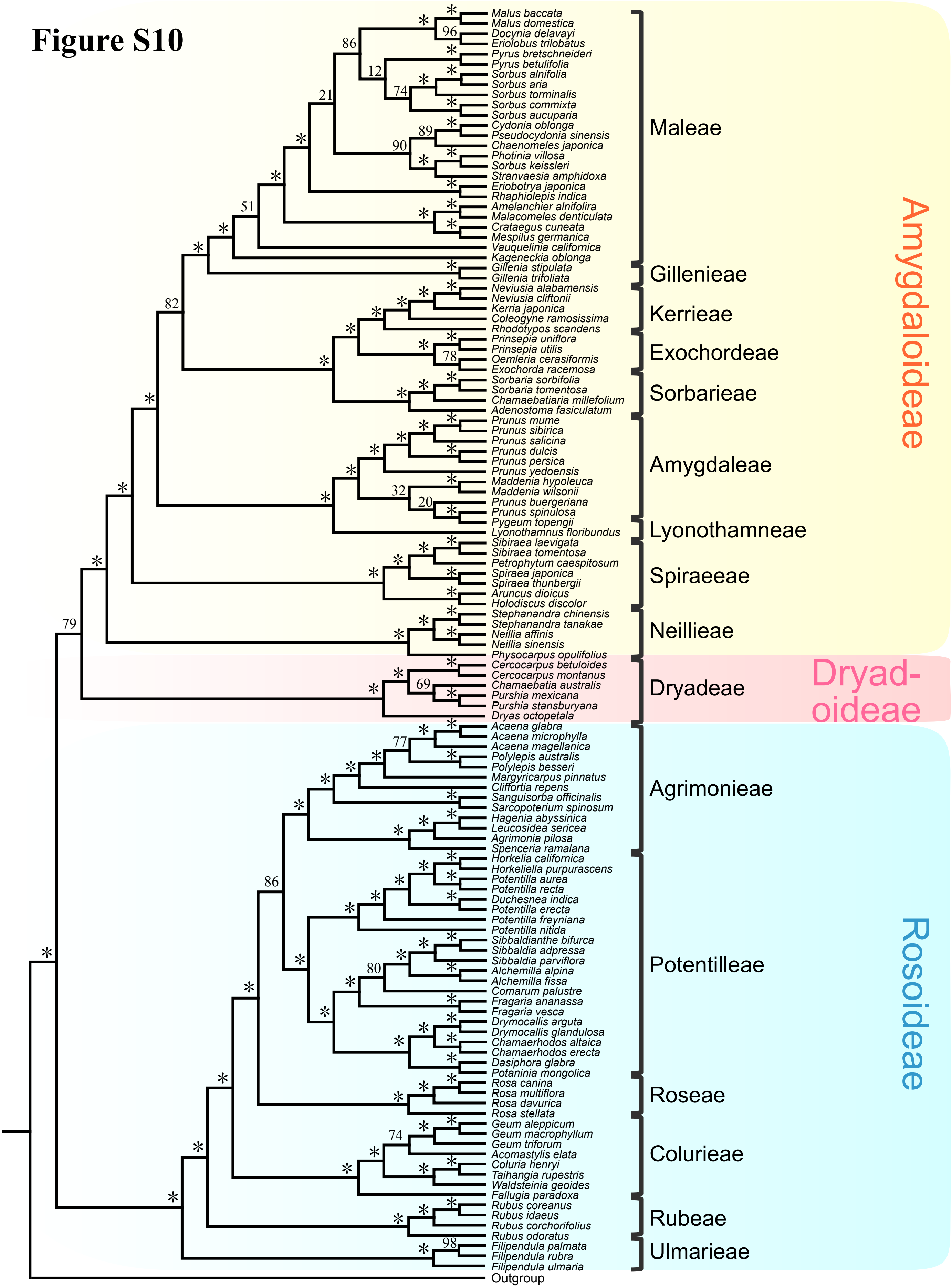

Figure S10. A phylogeny from coalescence analysis using 166 genes.

Numbers indicate values of support obtained by ASTRAL. Asterisks (*) indicate 100% support.

Figure S11

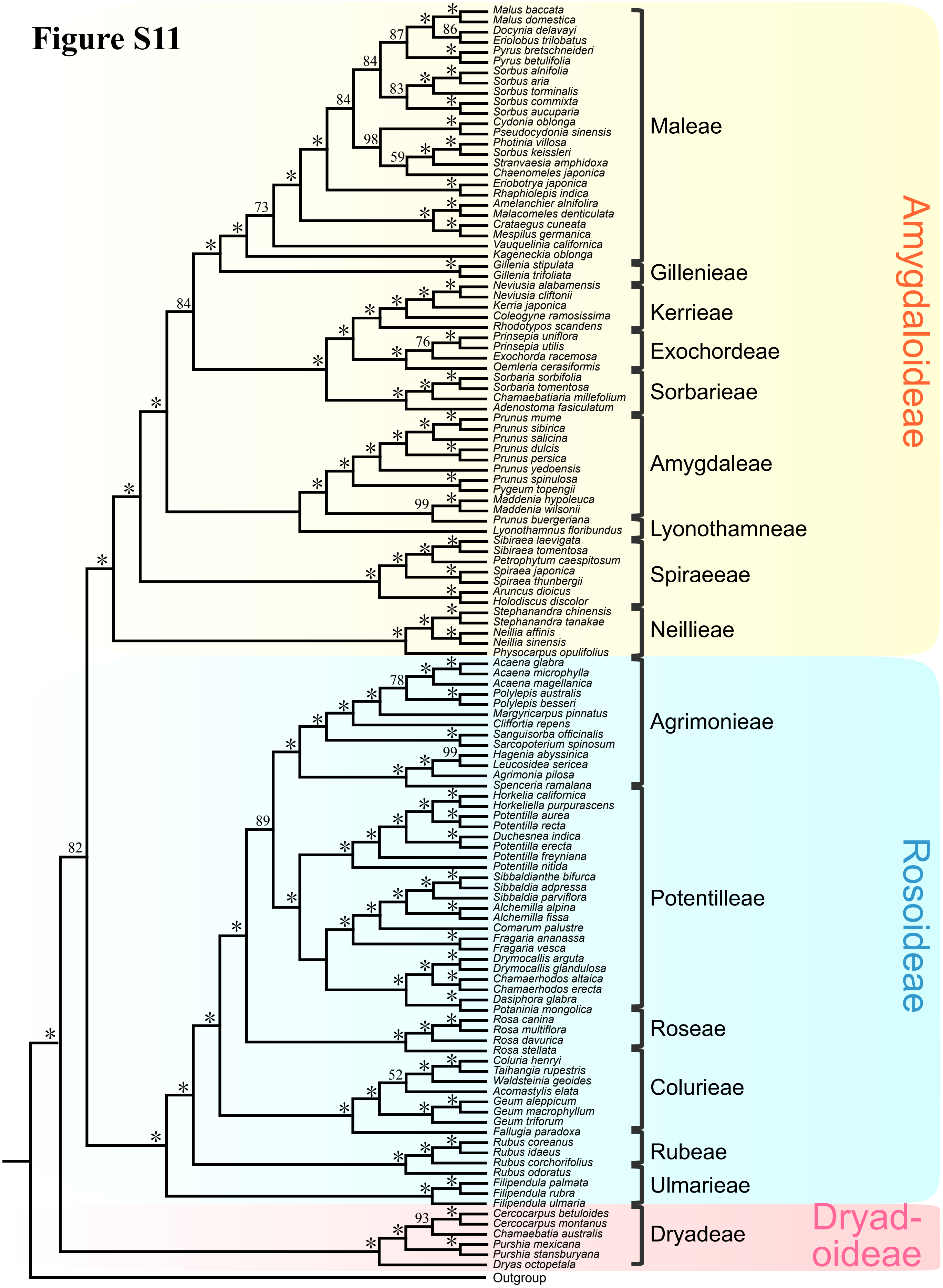

Figure S11. A phylogeny from coalescence analysis using 312 genes.

Numbers indicate values of support obtained by ASTRAL. Asterisks (*) indicate 100% support.

Figure S12

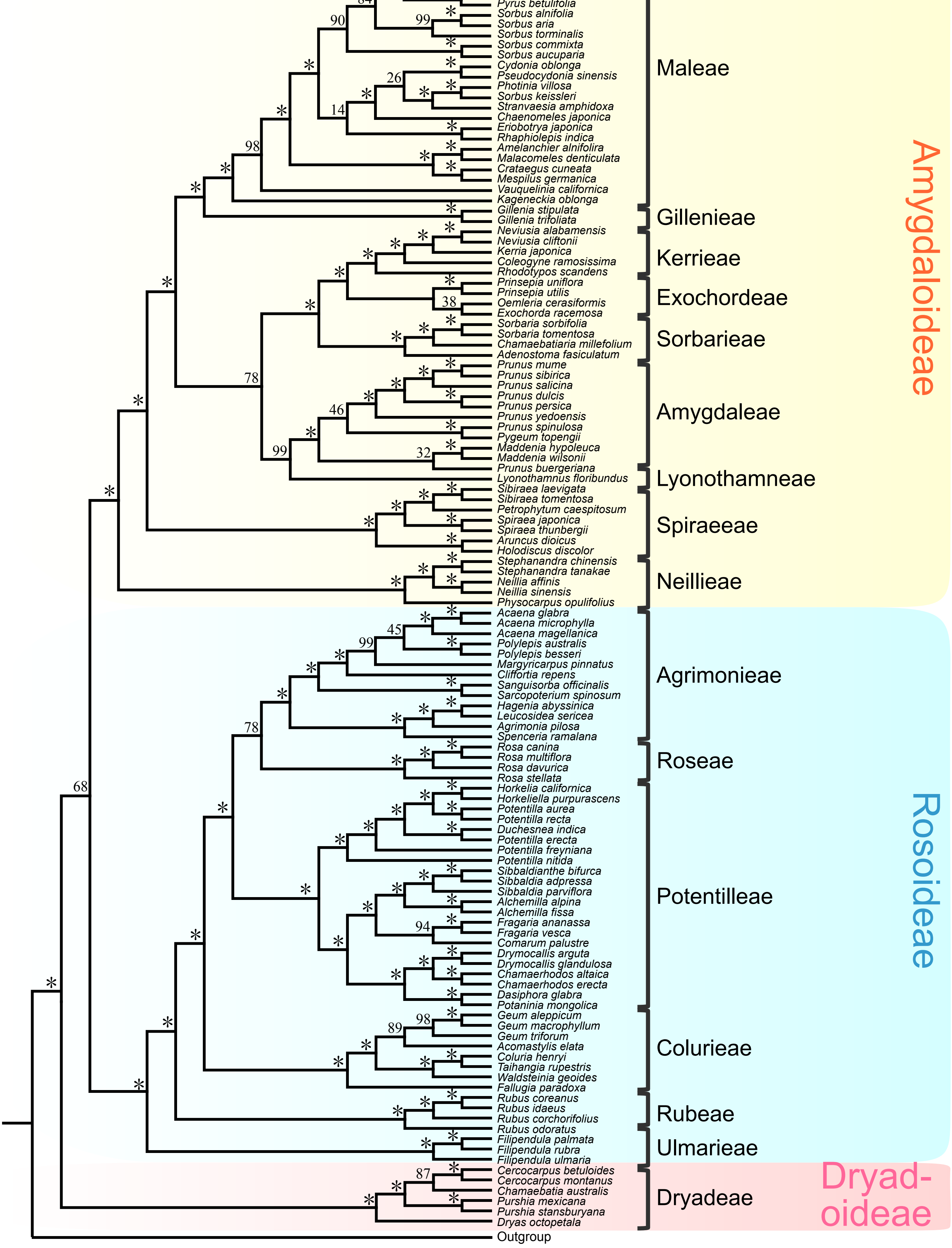

Figure S12. A phylogeny from coalescence analysis using 163 genes.

Numbers indicate values of support obtained by ASTRAL. Asterisks (*) indicate 100% support.

Figure S13

A

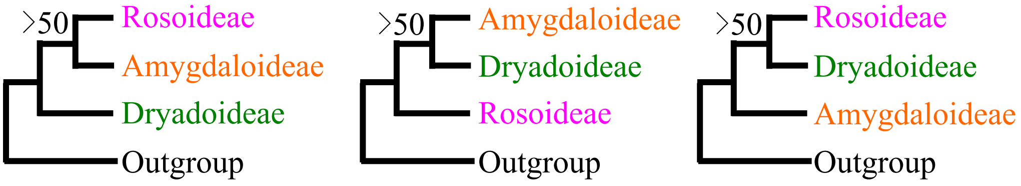

241 gene set	74	55	48
166 gene set	39	46	42
312 gene set	91	85	66
163 gene set	47	38	38

B

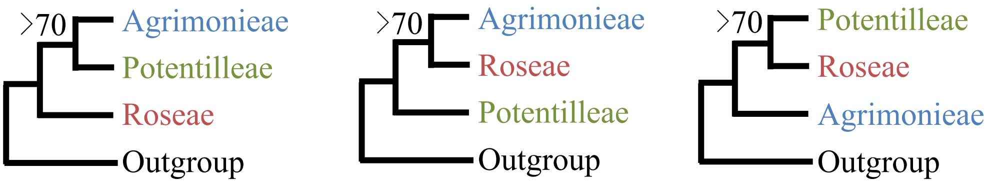

882 gene set	183	173	53
571 gene set	120	118	37
311 gene set	63	55	16
444 gene set	97	96	29
256 gene set	69	59	15
113 gene set	36	24	6
143 gene set	33	35	9
241 gene set	45	40	15
166 gene set	40	40	9
312 gene set	68	61	19
163 gene set	30	32	10

Figure S13. Numbers of single-gene trees supporting specific topologies.

A. Different relationships for the three subfamilies. B. Different positions of Roseae, relatitive to two other tribes. In each part, the topology on the left is consistent with that in Figure 1, while the other two are alternatives. The numbers below each topology represent the number of single-gene trees supporting the topology. In part B, the 311 gene set includes genes in the 882 gene set but not in the 571 gene set. The 143 gene set includes genes in the 256 gene set but not in the 113 gene set.

Figure S14

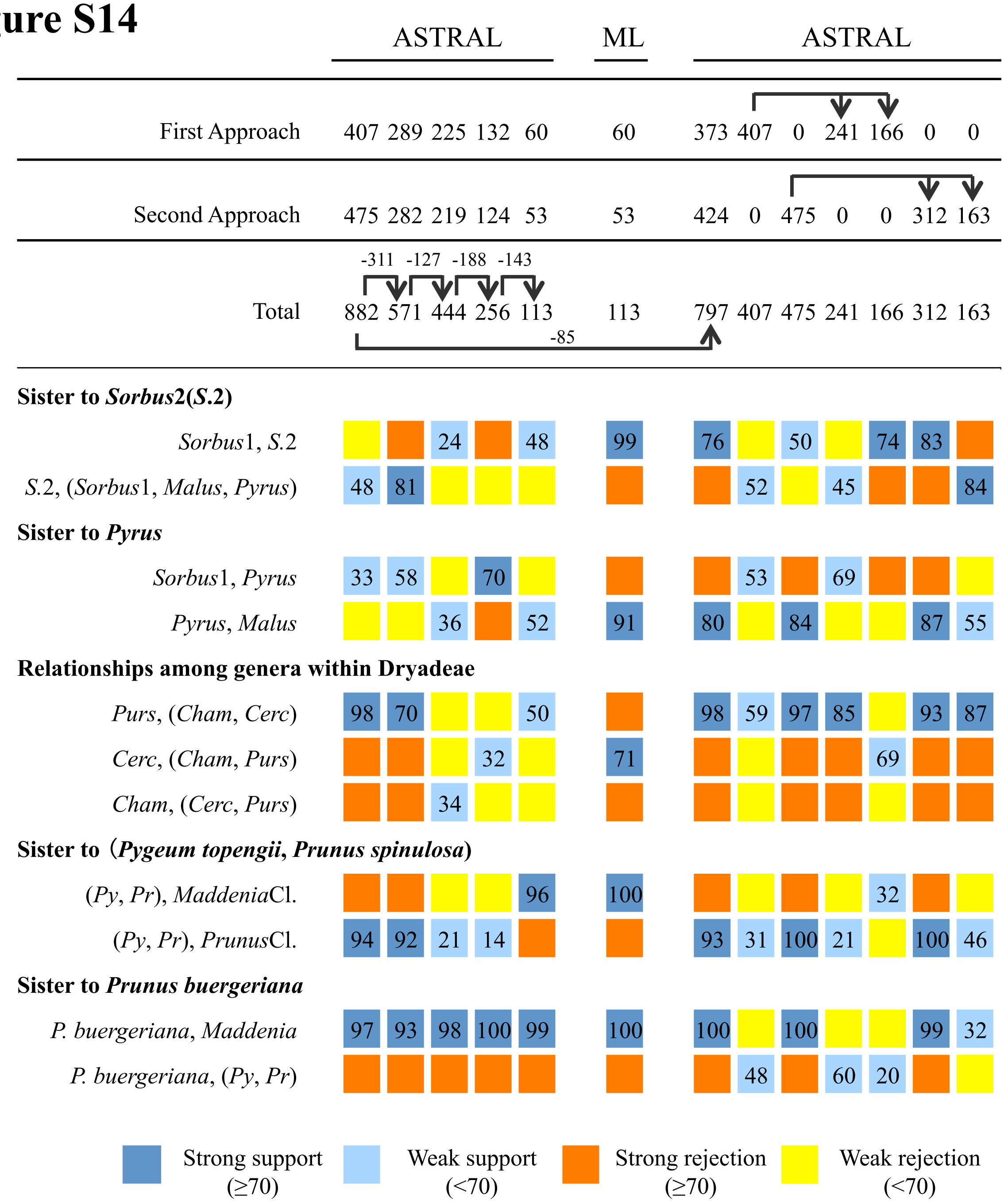

Figure S14. A summary of additional alternative topologies from twelve coalescence analyses results and one concatenation analysis.

At the top are phylogenetic methods and numbers of genes in various sets and relationships between gene sets (see Results, Materials and Methods, and Figure S24 for additional information). The column on the left indicates possible topologies. Designations: *Sorbus*1: *Sorbus alnifolia*, *Sorbus aria* and *Sorbus torminalis*. *Sorbus*2 (or *S.*2): *Sorbus commixta* and *Sorbus aucuparia*. *Purs*: *Purshia*. *Cham*: *Chamaebatia*. *Cerc*: *Cercocarpus*. *Py*: *Pygeum topengii*. *Pr*: *Prunus spinulosa*. *Maddenia*Cl.: a combined clade of *Maddenia hypoleuca*, *Maddenia wilsonii* and *Prunus buergeriana*. *Prunus*Cl.: a combined clade of *Prunus mume* and its five closest relatives (see Figure 1). Number in each square refers to values in support of a particular topology indicated in the left column. Strong support refers to support values of at least 70%. Weak support refers to values less than 70%. If there is a strong support for a topology in a particular node, other topologies at this node are strongly rejected. If there is a weak support for a topology in a particular node, other topologies at this node are weakly rejected.

Figure S15

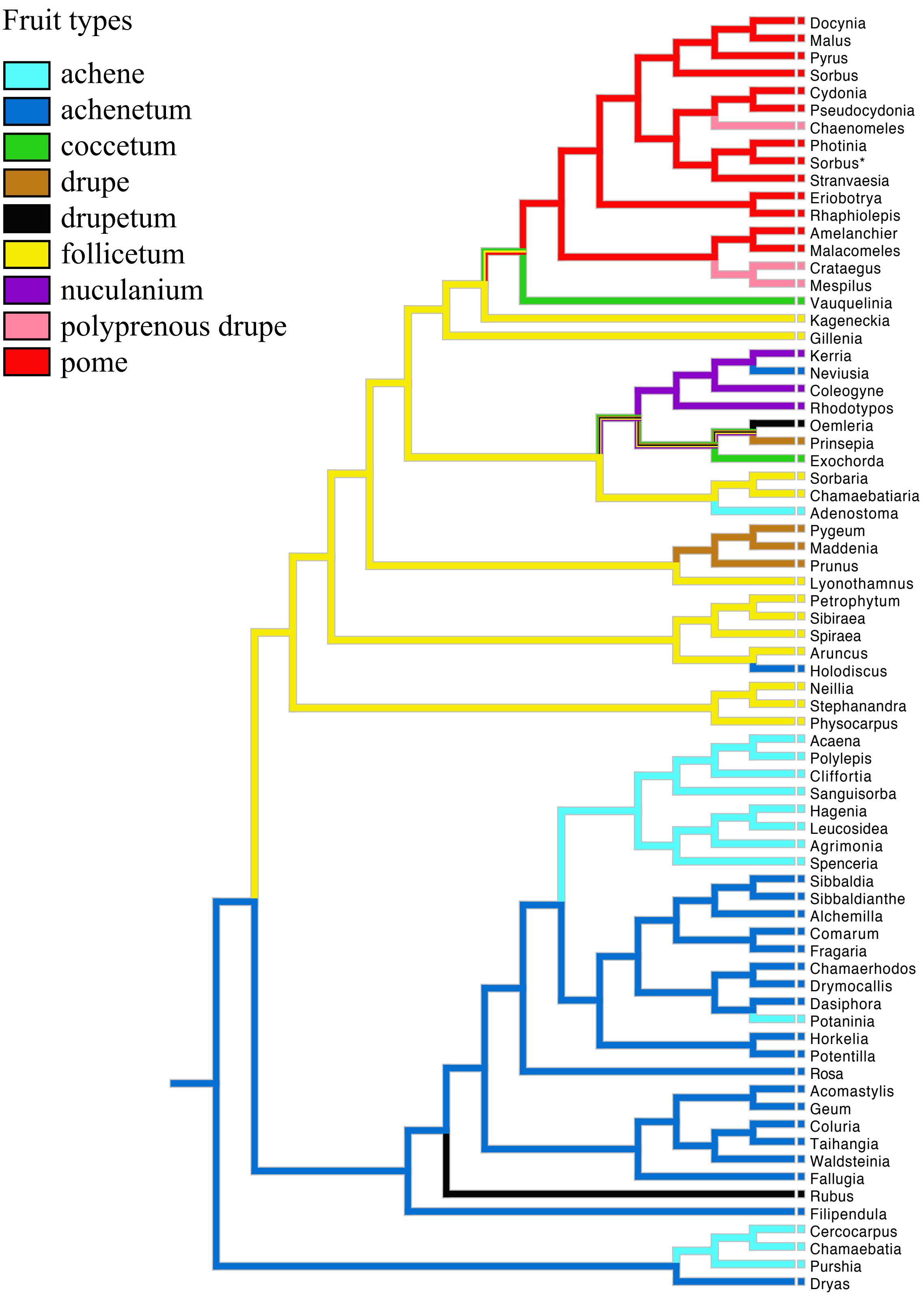

Figure S15. Reconstruction of ancestral fruit types in the context of the topology in Figure 1.

Figure S16

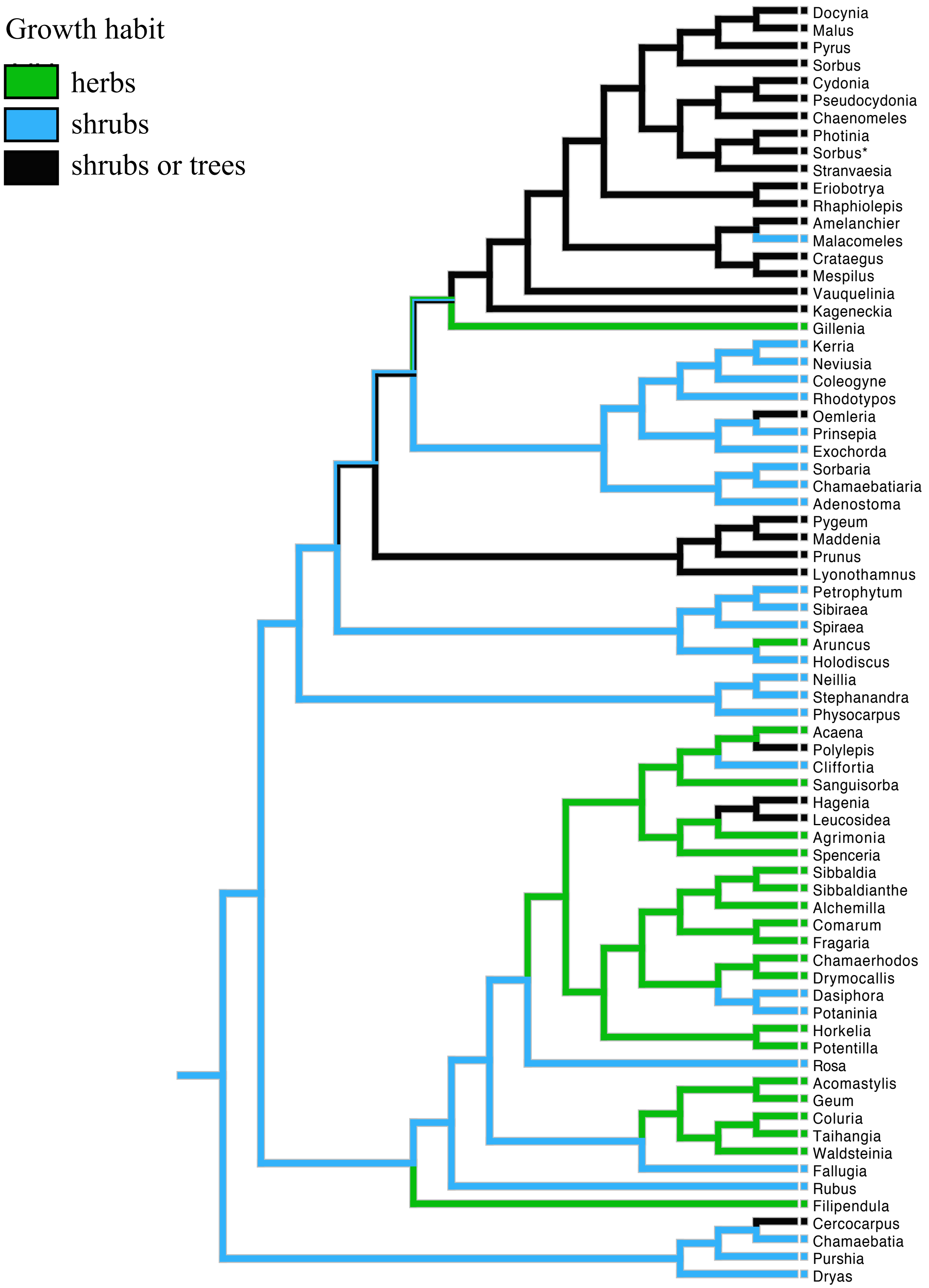

Figure S16. Reconstruction of ancestral growth habit in the context of the topology in Figure 1.

Figure S17

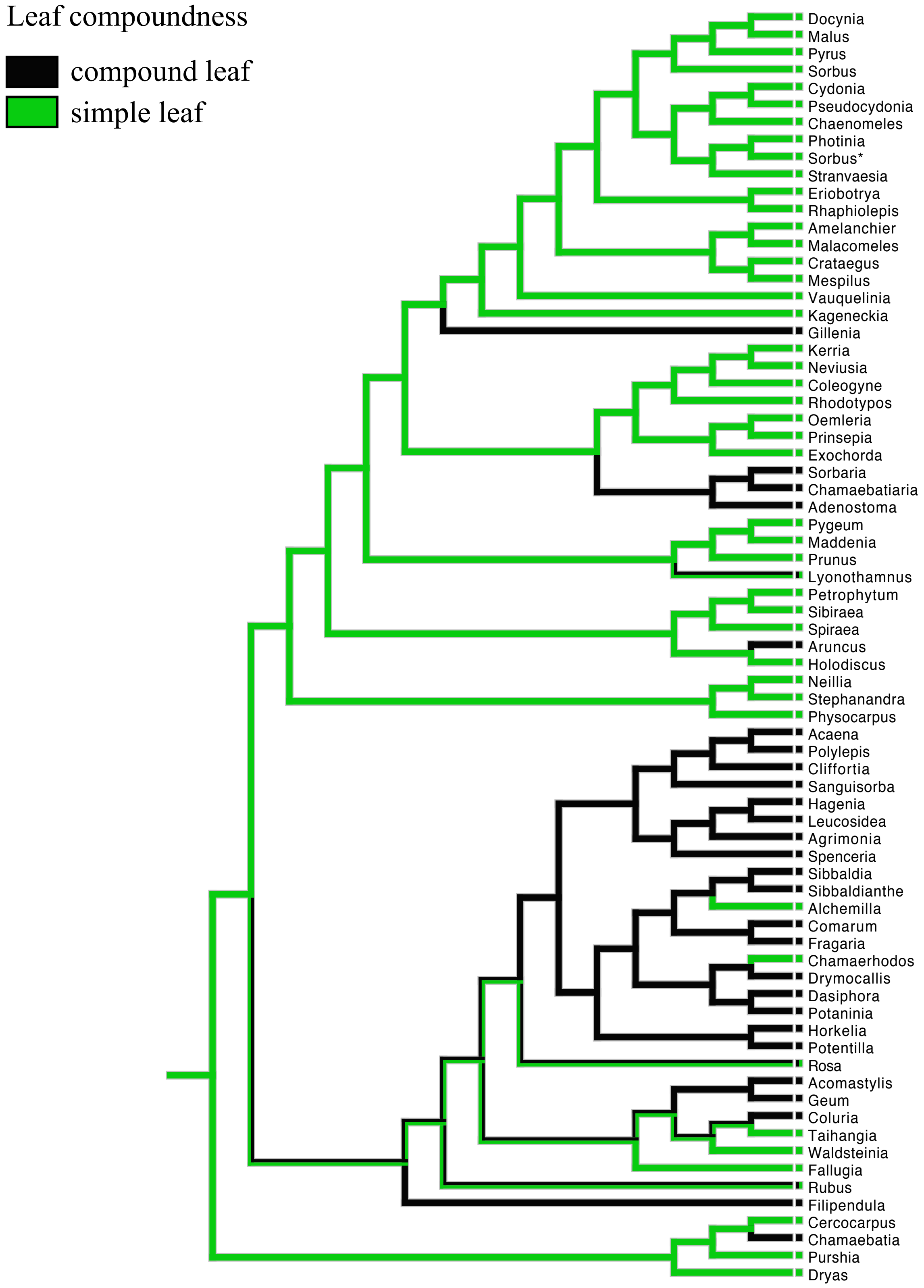

Figure S17. Reconstruction of ancestral character for leaf compoundness in the context of the topology in Figure 1.

Figure S18

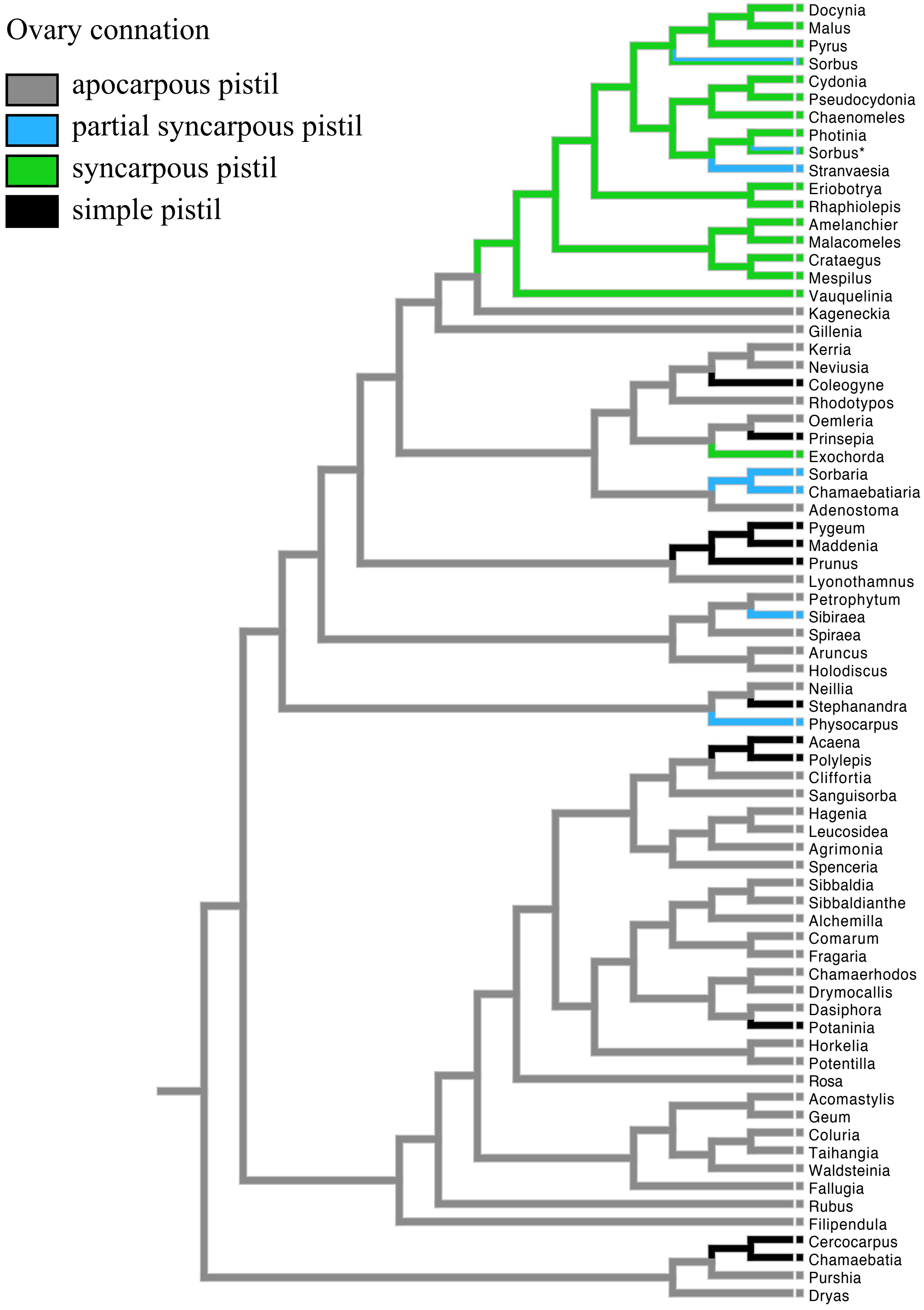

Figure S18. Reconstruction of ancestral character of ovary connation in the context of the topology in Figure 1.

Figure S19

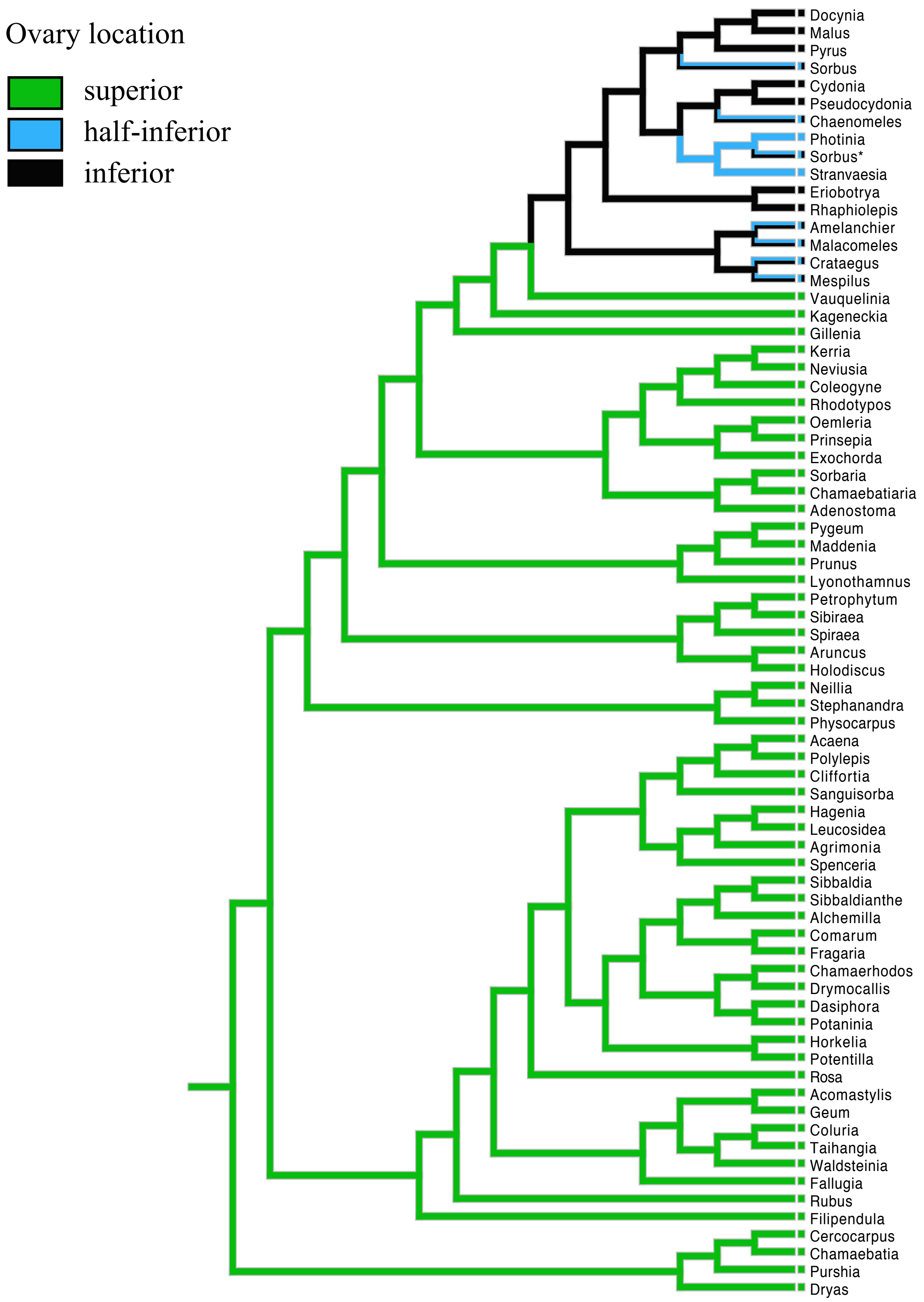

Figure S19. Reconstruction of ancestral ovary location in the context of the topology in Figure 1.

Figure S20

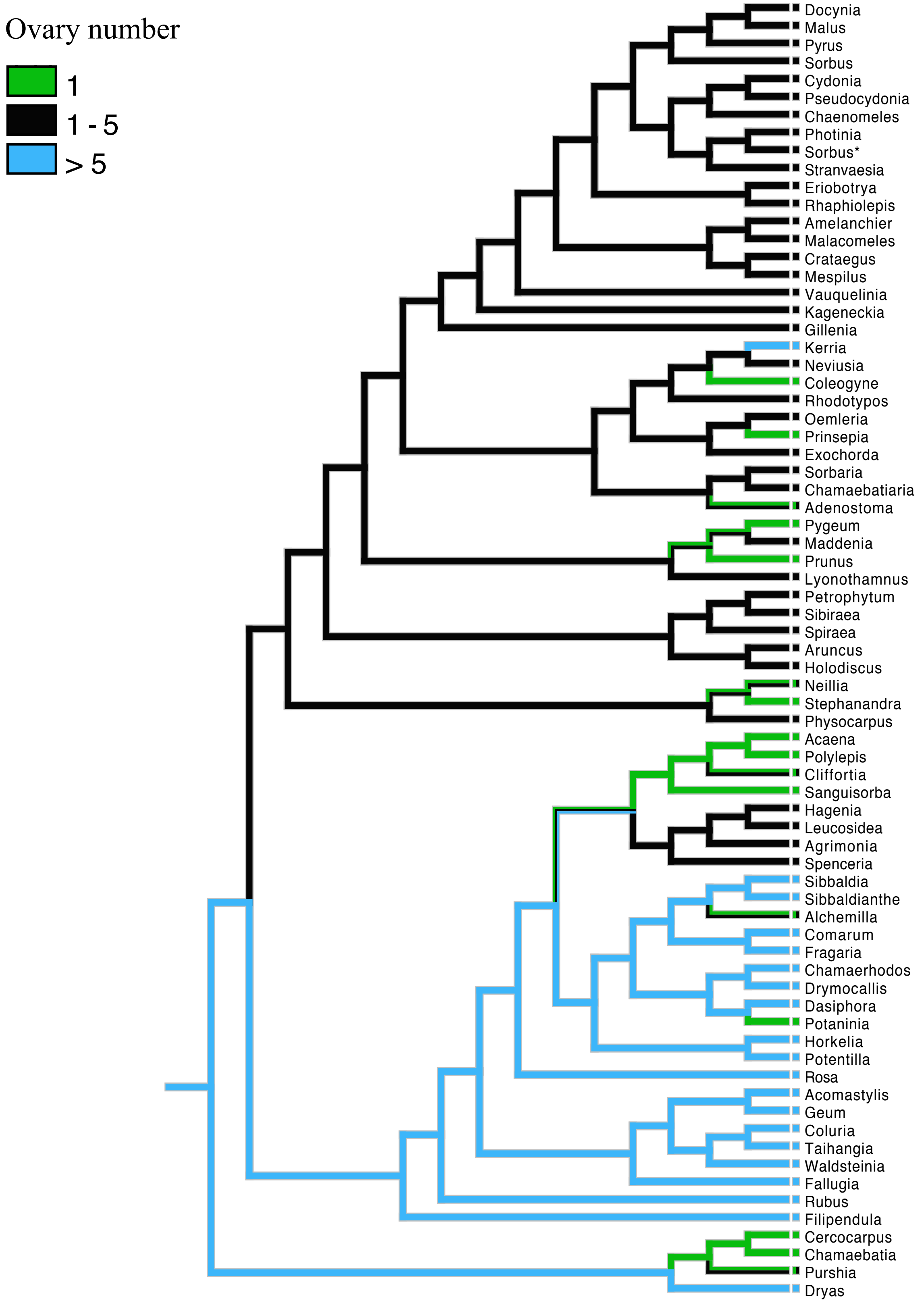

Figure S20. Reconstruction of ancestral ovary number in the context of the topology in Figure 1.

Figure S21

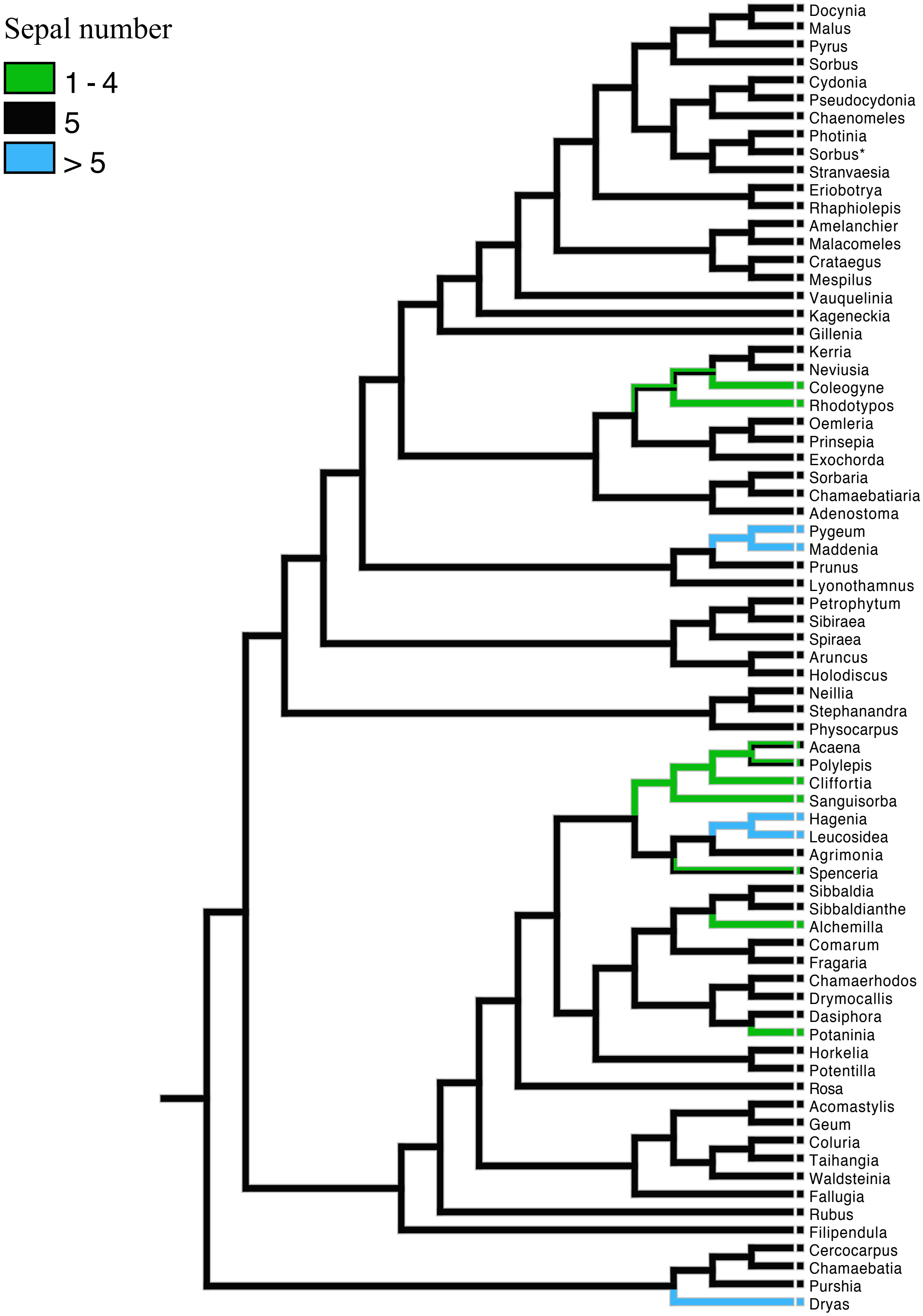

Figure S21. Reconstruction of ancestral sepal number in the context of the topology in Figure 1.

Figure S22

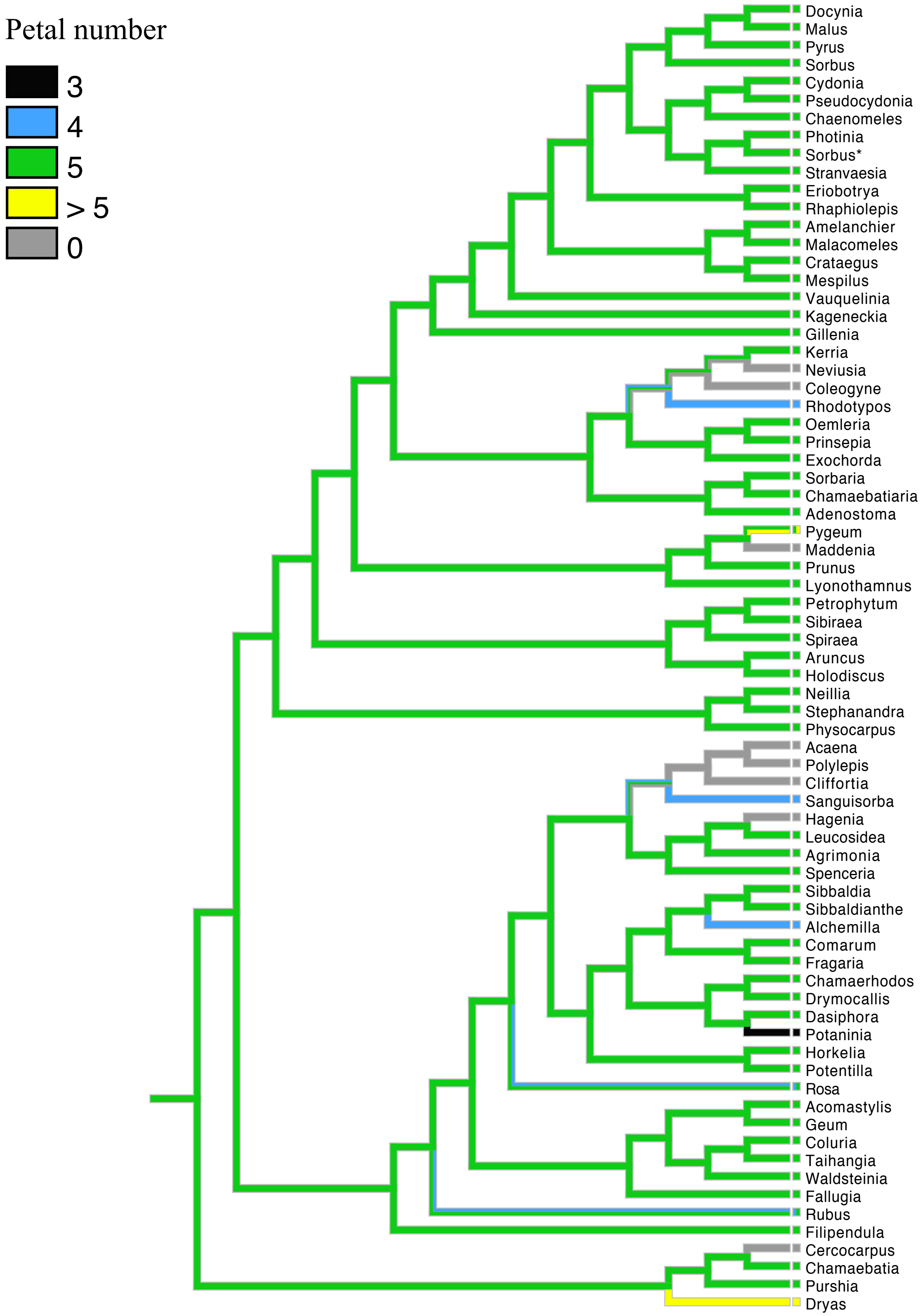

Figure S22. Reconstruction of ancestral petal number in the context of the topology in Figure 1.

Figure S23

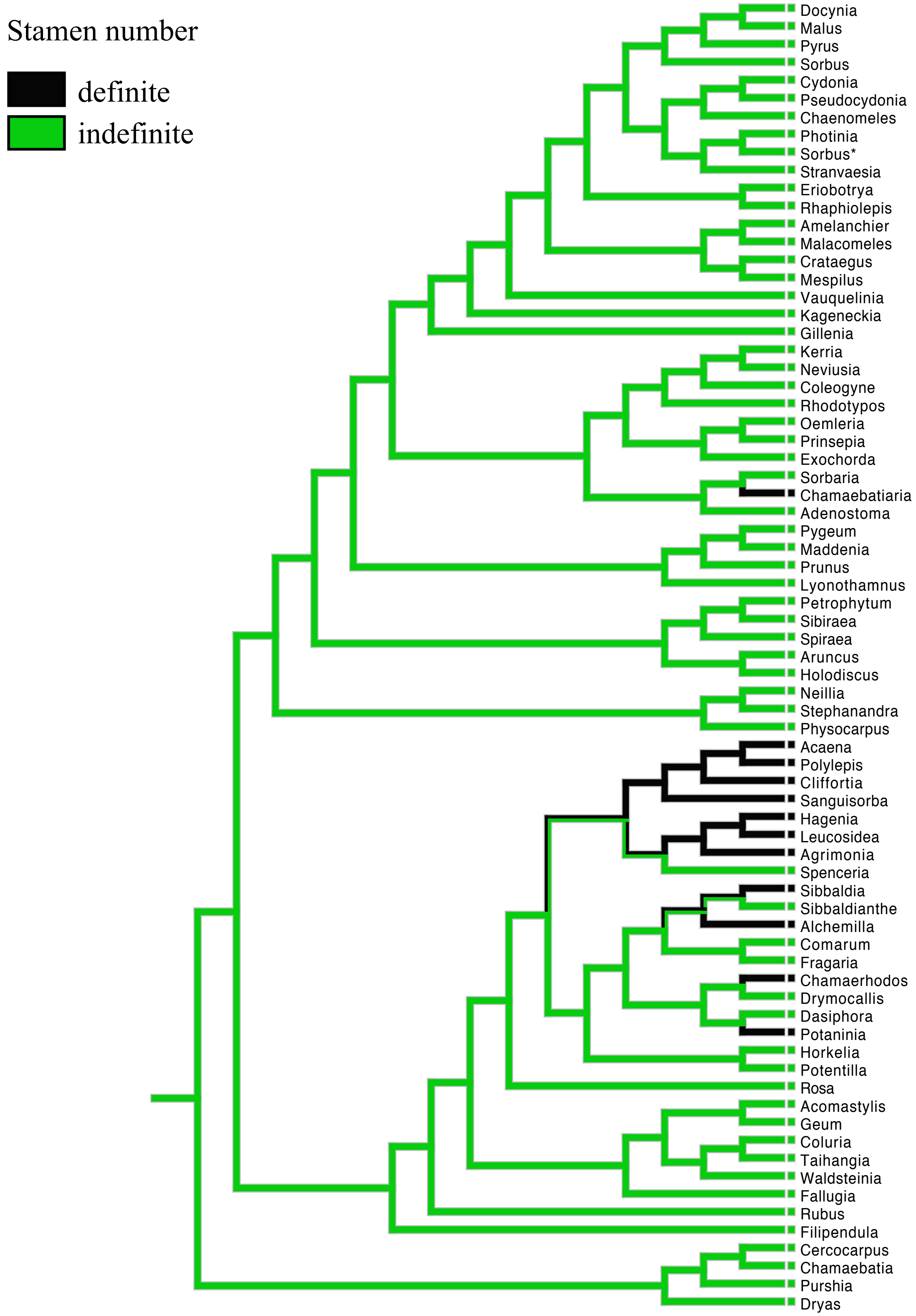

Figure S23. Reconstruction of ancestral stamen number in the context of the topology in Figure 1.

Figure S24

A

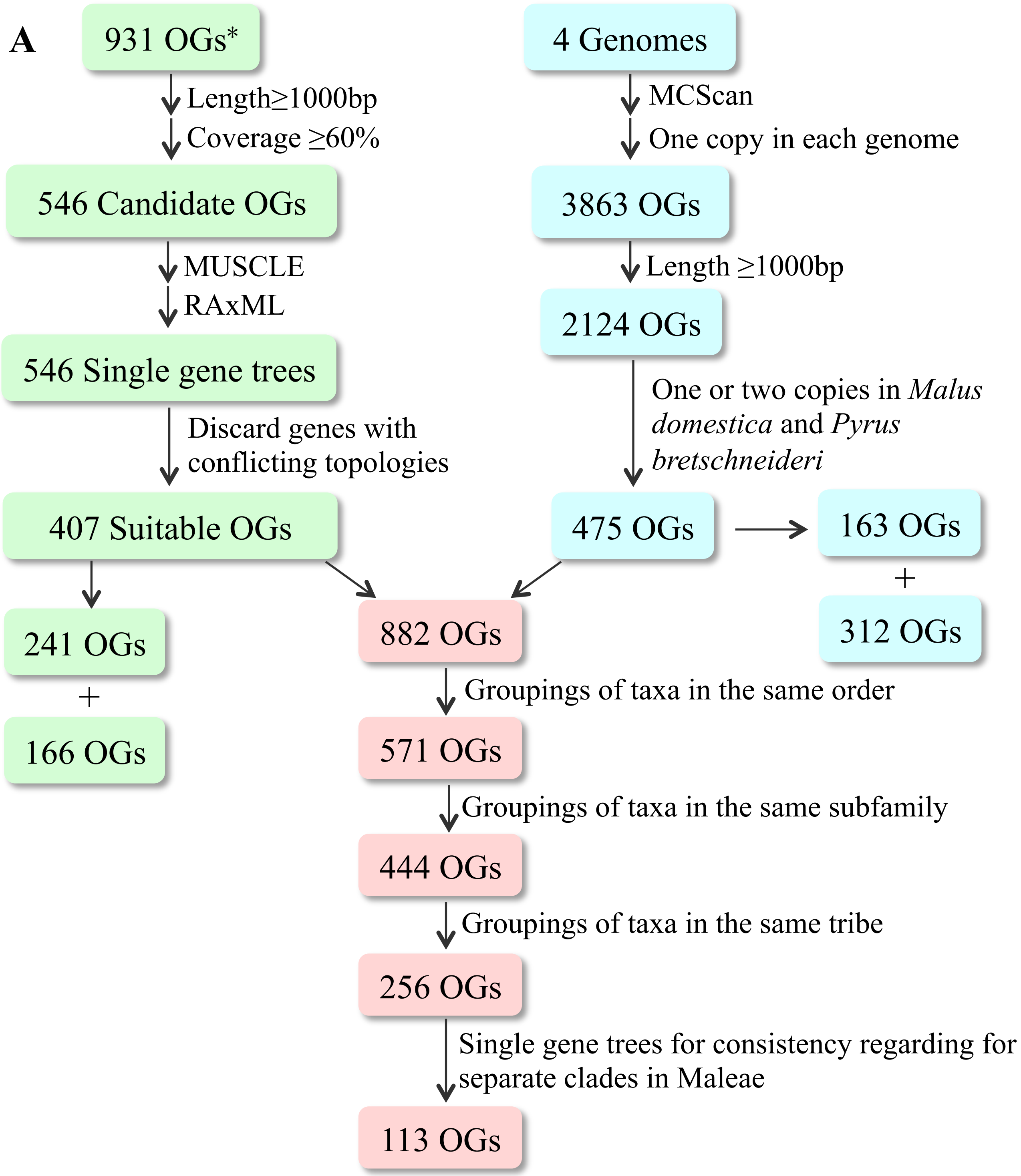

B

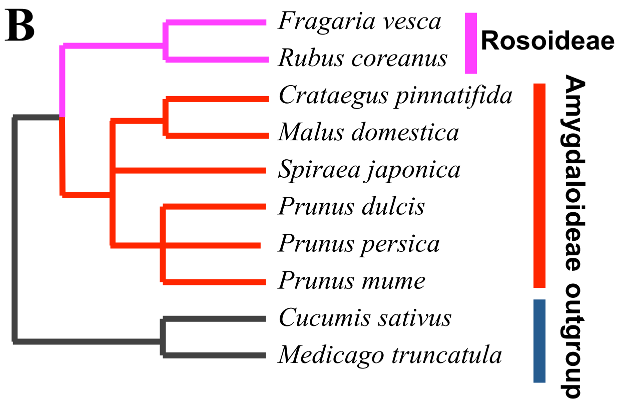

Figure S24. A: A flowchart of identification for low copy candidate orthologous genes. B: Established relationships of eight Rosaceae species.

*931 OGs were retrieved from Zeng et al. 2014
