# Supplementary material for: Evolution of Rosaceae Fruit Types Based on Nuclear Phylogeny in the Context of Geological Times and Genome Duplication
Source: Mol Biol Evol. 2016 Nov 17;34(2):262–81. doi: 10.1093/molbev/msw242 (PMC5400374; doi:10.1093/molbev/msw242)
Supplement: Supplementary Data [file msw242_Supp.zip › Table S6-fossils.pdf]

Table S6. Fossil constraints implemented in this study.

Numbers of nodes corresponds to those in Figure 3.

Node	Fossil name	Clade	Constraint age (My)	References
1	*Amelanchier peritula* / *Amelanchier scudderi*	Stem *Amelanchier*	33.9	Cockerell (1911); MacGinitie (1953)
2	*Vauquelinia comptonifolia*	Stem *Vauquelinia*	40.4	MacGinitie (1969)
3	*Neviusia* sp.	Stem *Neviusia*	48.6	Mathews (1964); Wehr and Hopkins (1994); DeVore et al. (2004)
4	*Oemleria janhartfordae*	Stem *Oemleria*	48.6	Wolfe et al. (2003); Greenwood et al. (2005); DeVore and Pigg (2007); Benedict et al. (2011)
5	*Prunus wutuensis*	Stem *Prunus*	55	Li et al. (2011)
6	*Holodiscus lisii*	Stem *Holodiscus*	34.1	Schorn (1998); McIntosh and Chapin (2004)
7	*Spiraea* sp.	Stem *Spiraea*	48.6	Mathews (1964); Wehr and Hopkins (1994); DeVore and Pigg (2007)
8	*Fragaria* sp.	Stem *Fragaria*	2.96	Matthews and Ovenden (1990); Matthews et al. (2003)
9	*Acaena* sp.	Stem *Acaena*	37.2	Zetter et al. (1999)
10	*Rosa germerensis*	Stem *Rosa*	48.6	Edelman (1975)
11	*Rubus acutiformis*	Stem *Rubus*	41.3	Chandler (1963)
12	*Cercocarpus myricaefolius*	Stem *Cercocarpus*	34.07	MacGinitie (1953); Evanoff et al. (2001)
13	*Prunus aspensis*	Stem Rosaceae	100.5	Peppe et al. (2008).
14	*Zizyphus* sp.	Stem Rhamnaceae	93.9	Spicer et al. (2002)
15	Moraceae indet.	Stem Moraceae	113	Vega et al. (2006)

16	*Quercus wardiana*	Stem Fagales	100.5	Ward (1899)
17	Normapolles complex	stem group core-Fagales	96.6	Magallón and Castillo (2009); Góczán et al. (1967); Pacltová (1981)
18	*Microaltingia apocarpela*	stem group Altingiaceae	89.3	Zhou et al. (2001)
19	Tricolpate pollen	crown group Eudicots	125	Doyle et al. (1977); Hughes and McDougall (1990); Doyle and Hotton (1991)

References

Benedict JC, DeVore ML, Pigg KB. 2011. *Prunus* and *Oemleria* (Rosaceae) flowers from the late early Eocene Republic flora of northeastern Washington State, U.S.A. Int J Plant Sci. 172:948-958.

Chandler MEJ. 1963. The Lower Tertiary Floras of Southern England. III. . Flora of the Bournemouth Beds; The Boscombe, and the Highcliff Sands. London: British Museum (Natural History). p. 1-169.

Cockerell TDA. 1911. Fossil Insects from Florissant, Colorado. Bulletin American Museum of Natural History. 30:71-82.

DeVore LM, Pigg BK. 2007. A brief review of the fossil history of the family Rosaceae with a focus on the Eocene Okanogan Highlands of eastern Washington State, USA, and British Columbia, Canada. Plant Syst Evol. 266:45-57.

DeVore ML, Moore SM, Pigg KB, Wehr WC. 2004. Fossil *Neviusia* leaves (Rosaceae: Kerriae) from the lower-middle Eocene of southern British Columbia. Rhodora. 106:197-209.

Doyle J, Biens P, Doerenkamp A, Jardiné S. 1977. Angiosperm pollen from the pre-Albian Lower Cretaceous of equatorial Africa. Bulletin Centres Recherches Exploration-Production Elf-Aquitaine. 1:451-473.

Doyle J, Hotton C. 1991. Diversification of early angiosperm pollen in a cladistic context. In: Blackmore S, Barnes S, editors. Pollen and Spores: Pattern of Diversification. Oxford, UK: Clarendon Press. p. 169-195.

Edelman DW. 1975. The Eocene Germer Basin Flora of South-Central Idaho. [M.S. Thesis]. [Moscow]: University of Idaho.

Evanoff E, McIntosh WC, Murphey PC. 2001. Fossil flora and stratigraphy of the Florissant Formation, Colorado. Proc Denver Museum Nat Sci. 4:1-16.

Góczán F, Groot J, Krutzsch W, Pacltová B. 1967. Die Gattungen des "Stemma normapolles Pflug 1953b" (Angiospermae). Paläont. Abh.

2B:429-539.
Greenwood DR, Archibald SB, Mathewes RW, Moss PT. 2005. Fossil biotas from the Okanagan Highlands, southern British Columbia and northeastern Washington State: climates and ecosystems across an Eocene landscape. Canadian Journal of Earth Sciences. 42:167-185.
Hughes N, McDougall A. 1990. Barremian-Aptian angiospermoid pollen records from southern England. Review of Palaeobotany and Palynology. 65:145-151.
Li Y, Smith T, Liu C-J, Awasthi N, Yang J, Wang Y-F, Li C-S. 2011. Endocarps of *Prunus* (Rosaceae: Prunoideae) from the early Eocene of Wutu, Shandong Province, China. Taxon. 60:555-564.
MacGinitie HD. 1969. The Eocene Green River Flora of northwestern Colorado and northeastern Utah. University of California Publications in Geological Sciences. 83:1-140.
MacGinitie HD. 1953. Fossil plants of the Florissant Beds Colorado. Carnegie Institution of Washington, Contributions to Paleontology. 599:1-198.
Magallón S, Castillo A. 2009. Angiosperm diversification through time. Am J Bot. 96:349-365.
Mathews WH. 1964. Potassium-Argon age determination of Cenozoic volcanic rocks from British Columbia. Geological Society of America Bulletin. 75:465-468.
Matthews JV, Ovenden LE. 1990. Late Tertiary plant macrofossils from localities in arctic/subarctic North America: a review of the data. Arctic. 43:364-392.
Matthews JV, Westgate JA, Ovenden L, Carter LD, Fouch T. 2003. Stratigraphy, fossils, and age of sediments at the upper pit of the Lost Chicken gold mine: new information on the late Pliocene environment of east central Alaska. Quatern Res. 60:9-18.
McIntosh WC, Chapin CE. 2004. Geochronology of the central Colorado volcanic field. New Mexico Bureau of Geology & Mineral Resources, Bulletin. 160:205-237.
Pacltová B. 1981. The evolution and distribution of normapolles pollen during the Cenophytic. Rev Palaeobot Palynol. 35:175-208.
Peppe DJ, Hickey LJ, Miller IM, Green WA. 2008. A morphotype catalogue, floristic analysis and stratigraphic description of the Aspen Shale flora (Cretaceous–Albian) of Southwestern Wyoming. Bulletin of the Peabody Museum of Natural History. 49:181-208.
Schorn HE. 1998. *Holodiscus lisii* (Rosaceae): a new species of ocean spray from the late Eocene Florissant Formation, Colorado, USA. . PaleoBios. 18:21–24.

Spicer RA, Ahlberg A, Herman AB, Kelley SP, Raikevich MI, Rees PM. 2002. Palaeoenvironment and ecology of the middle Cretaceous Grebenka flora of northeastern Asia. Palaeogeogr, Palaeoclimatol, Palaeoecol. 184:65-105.
Vega FJ, García-Barrera P, Perrilliat MdC, Coutiño MA, Marino-Pérez R. 2006. El Espinal, a new plattenkalk facies locality from the Lower Cretaceous Sierra Madre Formation, Chiapas, southeastern Mexico. Revista mexicana de ciencias geológicas. 23:323-333.
Ward LF. 1899. Cretaceous flora of the Black Hills. The Cretaceous formation of the Black Hills as as indicated by the fossil plants. Washington, USA: Government Printing Office.
Wehr WC, Hopkins DQ. 1994. The Eocene orchards and gardens of Republic, Washington. Washington Geology. 22:27–34.
Wolfe JA, Gregory-Wodzicki KM, Molnar P, Mustoe G. 2003. Rapid uplift and then collapse in the Eocene of the Okanagan? evidence from paleobotany. Geological Association of Canada–Mineralogical Association of Canada–Society of Economic Geologists, Joint Annual Meeting; Vancouver, Canada. Abstracts 28:p. abstract 533.
Zetter R, Hofmann CC, Draxler I, de Cabrera JD, Vergel MM, Vervoorst F. 1999. A rich Middle Eocene microflora at Arroyo de los Mineros, near Cañadón Beta, NE Tierra del Fuego Province, Argentina. Abhandlungen der Geologischen Bundesanstalt. 56:436-460.
Zhou Z, Crepet W, Nixon K. 2001. The earliest fossil evidence of the Hamamelidaceae: Late Cretaceous (Turonian) inflorescences and fruits of Altingioideae. Am J Bot. 88:753-766.
